# Fat-Free Mass Index, Visceral Fat Level, and Muscle Mass Percentage Better Explain Deviations From the Expected Value of Aortic Pressure and Structural and Functional Arterial Properties Than Body Fat Indexes

**DOI:** 10.3389/fnut.2022.856198

**Published:** 2022-04-29

**Authors:** Mariana Gómez-García, Juan Torrado, María Pereira, Daniel Bia, Yanina Zócalo

**Affiliations:** ^1^Departamento de Educación Física y Salud, Instituto Superior de Educación Física, Universidad de la República, Montevideo, Uruguay; ^2^CUiiDARTE-Movimiento, Actividad, Salud (CUiiDARTE-MAS), Comisión Sectorial de Investigación Científica, Universidad de la República, Montevideo, Uruguay; ^3^Department of Internal Medicine, Jacobi Medical Center, Albert Einstein College of Medicine, New York, NY, United States; ^4^Department of Obstetrics and Gynecology, BronxCare Hospital Center a Clinical Affiliate of Mt Sinai Health Systems and Academic Affiliate of Icahn School of Medicine, New York, NY, United States; ^5^Departamento de Fisiología, Facultad de Medicina, Centro Universitario de Investigación, Innovación y Diagnóstico Arterial (CUiiDARTE), Universidad de la República, Montevideo, Uruguay

**Keywords:** aortic pressure, arterial stiffness, bioelectrical impedance analysis, body composition assessment techniques, cardiovascular diagnosis, cardiovascular research, epidemiological research, intima-media thickness

## Abstract

Bioelectrical impedance analysis (BIA)-derived indexes [e.g., fat (FMI) and fat-free mass indexes (FFMI), visceral fat level (VFL)] are used to characterize obesity as a cardiovascular risk factor (CRF). The BIA-derived index that better predicts arterial variability is still discussed.

**Aims:**

To determine: (1) the association of classical [weight, height, body mass index (BMI), basal metabolic rate (BMR)] and BIA-derived indexes, with arterial properties deviations from expected values (arterial z-scores); (2) maximum arterial variations attributable to BIA-derived indexes; (3) whether the composition of total body, trunk and/or limbs is most closely associated with arterial variations.

**Methods:**

Hemodynamic, structural, and functional parameters of different histological types of arteries were assessed (*n* = 538, 7–85 years). Classical and BIA-derived indexes [fat mass and percentage, FMI, VFL, muscle mass percentage (PMM), FFMI, and percentage] were measured (mono- and multi-segmental devices). Arterial z-scores were obtained using age-related equations derived from individuals not-exposed to CRFs (*n* = 1,688).

**Results:**

First, regardless of the classical index considered, the associations with the arterial properties showed a specific hierarchy order: diameters and local stiffness > aortic and brachial blood pressure (BP) > regional stiffness. Second, all the associations of FMI and FFMI with z-scores were positive. Third, FFMI exceeded the association obtained with BMI and BMR, considering structural z-scores. In contrast, FMI did not exceed the association with z-scores achieved by BMI and BMR. Fourth, regardless of CRFs and classical indexes, arterial z-scores would be mainly explained by FFMI, VFL, and PMM. Fifth, regardless of the body-segment considered, the levels of association between FMI and z-scores did not exceed those found for classic and FFMI. Total fat mass and trunk indexes showed a greater strength of association with z-scores than the FMI of limbs. Sixth, compared to lower limb FFMI indexes, total and upper limbs FFMI showed higher levels of association with z-scores.

**Conclusions:**

FFMI (but not FMI) exceeded the strength of association seen between BMI or BMR and structural z-scores. Regardless of the body segment analyzed, the associations between FMI and z-scores did not exceed those found with classic and FFMI. Arterial z-scores could be independently explained by FFMI, VFL, and PMM.

## Introduction

The rate of progression of structural and functional arterial disease is directly associated with the exposure to an increasing number of cardiovascular risk factors (CRFs) (1, 2). In Latin America, as well as in all western societies, overweight and obesity represent a major public health concern, which affects virtually all age groups of different socioeconomic status (3, 4). Obesity has the particularity to increase the risk of other CRFs such as diabetes and hypertension, all of which further accelerate the development of arterial disease (5). Over the past years, there has been significant efforts of the medical community to reduce the obesity, which resulted in different countries to introduce policies aimed at obesity prevention, intensification of the control of associated CRFs, and early arterial disease screening (i.e., using non-invasive arterial evaluation) (6). Furthermore, there has been growing interest in trying to better understand the association between body composition and early arterial changes and/or disease by investigating, which is the best approach to characterize both body composition indexes [using fast, simple, non-invasive, and easily repeatable analyses such as bioelectrical impedance analysis (BIA)] and arterial impairment among subjects (7). Despite the fact that overweight and obesity have been associated with worse cardiovascular status and disease prognosis, classic indexes used to characterize abnormal or excessive fat accumulation [e.g., body weight (BW) or body mass index (BMI)] have shown predictive limitations (7). For instance, BMI does not consider fat distribution and may not indicate adequately fat content. In this regard, it could overestimate the degree of adiposity in both individuals who are overweight but very muscular (e.g., athletes) and in older patients who have lost body height (BH) secondary to spine osteoporosis. On the other hand, BMI may underestimate adiposity in elder individuals who have lost muscle mass in association with aging (8). Undoubtedly, this could result in inaccurate cardiovascular risk prediction at the patient level.

Recently, BIA-derived body fat distribution and the use of new indexes, such as fat mass and fat-free mass indexes (FMI and FFMI) have been used to characterize overweight and obesity. These parameters have shown an acceptable accuracy in estimating several health outcomes compared with classical anthropometric indexes (9, 10). Moreover, several studies have reported that FMI and FFMI are trustworthy obesity markers and are associated with cardiovascular abnormalities such as increased arterial stiffness, carotid wall thickness, and blood pressure (BP) (9, 11, 12). Yet, it remains unclear which BIA-derived body composition parameter better predicts structural, functional, and hemodynamic arterial changes. Additionally, the whole-body BIA (total or mono-segmental) aimed at measuring body composition could not be sensitive enough to detect specific segmental changes [i.e., increase in central (trunk), but not in lower limb adiposity]. Consequently, the measurement of both, the total body composition (mono-segmental BIA) and different body segments (trunk, lower and upper limbs; multi-segmental BIA) could theoretically provide complementary information about the link between body composition characteristics and cardiovascular properties.

Recently, our group has shown that traditional and non-traditional CRFs present different levels of association with parameters of arterial structure and function of different arterial territories [i.e., different histological type of arteries: elastic (carotids), transitional (brachial), and muscular (femoral)], as well as with different hemodynamic properties (13–19). We found that the impact of different CRFs, including time in sedentary behavior and sleep time (13), low birth weight and catch-up growth (14), physical activity level (e.g., assessed using hip- and wrist-worn accelerometers) (15), high BP (17) and z-BMI (as a continuous variable) or obesity (18, 19) could differ, depending on the arterial parameter (structural vs. functional) or territory (central vs. peripheral, elastic, muscular or transitional) considered. To our knowledge, there are no studies to date that have comprehensively analyzed the association between classical anthropometric and BIA-derived body composition indexes (i.e., fat mass, fat-free mass) and arterial properties, considering (i) central and peripheral BP levels, (ii) carotid, femoral, and brachial diameters, and wall thickness, and (iii) regional and local arterial stiffness of different vascular territories. On the other hand, an important issue would be to identify to what extent BIA-derived indexes are associated with arterial properties, independently of the exposure to other CRFs (including classical anthropometric indexes). BIA-derived indexes showing an independent association could be the most useful to indicate the expected values of arterial parameters regardless of other individuals' characteristics (e.g., age, sex, high BP, BMI).

As in previous studies, for each analysis that included the arterial system, we analyzed the levels at which each arterial parameter deviates from the expected “optimal” value, accounting for the subject age (z-score) (13–15). The z-score describes the position of a subject-specific raw score in terms of its “distance” from the mean value in standard deviation units. For instance, a z-score for arterial stiffness equal to + 2 or −2 indicates that a particular individual has stiffness levels of two standard deviations above or below the expected value, respectively, for an age-matched healthy individual not exposed to traditional CRFs (13–15). Thus, our analysis focuses on identifying the extent to which classical anthropometric and BIA-derived indexes would explain the level of deviation of the arterial system from the values considered “optimal,” independently of other factors. In this way, it is possible to analyze whether the levels of the body composition indexes are able to explain the “deviations from normality,” and not simply whether they are associated with the levels of the cardiovascular variables, which are expected to vary with age.

In this context, the aims of this work were (in healthy children, adolescents, and adults):

- First (Aim 1), to characterize the level of association between (i) classical [BW, BH, BMI, basal metabolic rate (BMR)], (ii) fat mass, and (iii) fat-free mass indexes, and cardiovascular z-scores (considering hemodynamic, structural, and functional parameters; central and peripheral arteries).
- Second (Aim 2), to evaluate and compare classical anthropometric variables with fat mass and fat-free mass indexes (mono-segmental BIA-derived), as potential explanatory variables of cardiovascular z-scores levels.
- Third (Aim 3), to quantify the maximum variations in cardiovascular variables (*effect size*), which can be attributed to variations in BIA-derived indexes.
- Finally (Aim 4), to analyze whether fat and fat-free mass distribution analysis (multi-segmental BIA) is able to identify specific body regions (e.g., total body vs. trunk vs. upper limbs vs. lower limbs) that are significantly associated with cardiovascular z-scores.

## Materials and Methods

### Study Population

This study was carried out in the context of the Centro Universitario de Investigación, Innovación y Diagnóstico Arterial (CUiiDARTE) project (13–27). This includes data derived from community-based studies on demographic and anthropometric variables, exposure to CRFs, personal and family history of cardiovascular disease and data on hemodynamic, structural, and functional vascular parameters. From this database, 538 subjects with body composition measurements with single-frequency mono-segmental BIA device were selected (110 of whom were also evaluated with multi-frequency multi-segmental BIA) (Table 1). Additionally, a “Reference Group” (*n* = 1,688) was selected from the CUiiDARTE project database (*n* = 3,619) in order to quantify cardiovascular z-scores (15, 21–25). All procedures were conducted in agreement with the Declaration of Helsinki (1975 and reviewed in 1983), and the study protocol was approved by the Institution's Ethics Committee. In adults, written informed consent was obtained prior to the evaluation. In children and adolescents (<18 y), parents' written consent and children's assent were provided before the study.

**Table 1:** Characteristics of subjects evaluated by bioelectrical impedance analysis (OMRON HBF-514C device).

	**All (*****n*** **= 538)**				**Male (*****n*** **= 286)**				**Female (*****n*** **= 252)**			
	**MV**	**SD**	**Min**	**Max**	**MV**	**SD**	**Min**	**Max**	**MV**	**SD**	**Min**	**Max**
**Cardiovascular risk factors**												
Age (years)	29.70	18.17	7.00	85.79	32.55	19.02	7.00	75.00	26.46	16.61	11.00	85.79
Current smoker (%)	9				9.2				8.9			
Hypertension (%)	13				17.9				8.1			
Dyslipidemia (%)	16				17.1				13.8			
Diabetes (%)	2				3.2				1.6			
Obesity (%)	13				14.8				9.9			
History of CVD (%)	0				0				0			
Family History of CVD (%)	8				7.2				9.0			
On anti-hypertensive drug (%)	11				15.3				5.7			
On anti-HLD drug (%)	9				12.1				4.9			
On anti-diabetic drug (%)	3				3.9				2.0			
**Anthropometric indexes**												
Body Height (OM) (m)	1.68	0.10	1.21	1.96	1.74	0.08	1.21	1.96	1.60	0.06	1.32	1.75
Body Weight (OM) (kg)	69.53	17.09	27.80	134.7	76.88	16.88	27.80	134.7	61.34	13.17	40.20	120.0
BMI (OM) (kg/m^2^)	24.56	4.85	15.10	48.20	25.24	4.71	15.10	40.60	23.82	4.90	16.50	48.20
BMR (OM) (kcal)	1,544	276	1,044	2,392	1,736	209	1,178	2,392	1,323	152	1,044	1,994
**Body fat mass indexes**												
BFM (OM) (kg)	20.00	10.15	2.60	65.40	18.32	10.18	2.60	52.50	21.86	9.81	5.42	65.40
PBF (OM) (%)	27.97	10.34	5.40	68.00	22.21	8.60	5.40	68.00	34.35	8.13	11.00	59.40
FMI (OM) (kg/m^2^)	7.19	3.80	0.93	28.60	5.99	3.24	0.93	17.25	8.54	3.94	2.07	28.60
VFL (OM) (range: 1–30)	7.18	4.94	1.00	27.00	8.96	5.47	1.00	27.00	5.13	3.21	1.00	23.20
**Body fat-free mass indexes**												
FFM (OM) (kg)	49.88	12.22	24.70	97.25	59.39	8.52	24.70	97.25	39.33	4.72	27.97	62.43
PMM (OM) (%)	32.32	7.12	16.90	49.20	37.11	5.75	23.20	49.20	26.80	3.73	16.90	46.00
FFMI (OM) (kg/m^2^)	17.51	2.72	7.62	25.93	19.49	1.97	7.62	25.93	15.29	1.41	12.91	21.56
PFFM (OM) (%)	72.03	10.34	32.00	94.60	77.79	8.60	32.00	94.60	65.65	8.13	40.60	89.00
**Arterial structural parameters**												
L-CCA DD (mm)	7.11	0.90	5.43	10.81	7.29	0.87	5.55	10.81	6.55	0.79	5.43	8.65
R-CCA DD (mm)	7.13	0.84	5.37	10.40	7.31	0.83	5.62	10.40	6.57	0.60	5.37	7.85
L-CFA DD (mm)	8.28	1.38	5.21	11.86	8.71	1.22	5.51	11.86	6.93	0.94	5.21	8.91
R-CFA DD (mm)	8.33	1.39	5.04	12.79	8.72	1.24	5.33	12.79	7.12	1.12	5.04	9.19
BA DD (mm)	4.20	0.71	2.67	5.72	4.39	0.64	2.67	5.72	3.50	0.46	2.90	4.38
**Arterial functional parameters**												
L-CCA IMT (mm)	0.72	0.20	0.29	1.24	0.75	0.20	0.29	1.24	0.65	0.19	0.41	1.17
R-CCA IMT (mm)	0.72	0.20	0.36	1.56	0.74	0.20	0.36	1.56	0.64	0.16	0.41	0.98
L-CCA EM (mmHg)	992	393	315	2,129	1,042	397	316	2,129	833	345	348	1,482
L- CCA Beta	9.93	3.72	3.51	21.87	10.37	3.83	3.51	21.87	8.56	3.05	4.36	14.07
R-CCA EM (mmHg)	943	387	279	2,291	984	379	397	2,291	815	390	279	1,632
R- CCA Beta	9.53	3.73	3.67	22.28	9.88	3.74	4.37	22.28	8.40	3.54	3.67	16.27
L-CFA EM (mmHg)	1,234	476	417	2,494	1,293	485	462	2,494	1,052	407	417	2,103
L- CFA Beta	12.61	4.64	4.41	27.16	13.07	4.78	4.41	27.16	11.18	3.93	5.68	19.69
R-CFA EM (mmHg)	1,215	502	416	2,990	1,263	516	458	2,990	1,066	434	416	2,181
R- CFA Beta	12.32	4.69	5.09	26.90	12.68	4.87	5.09	26.90	11.20	3.99	5.46	21.77
BA ME (mmHg)	1,471	827	346	3,792	1,592	833	408	3,792	1,025	641	346	2,902
BA Beta	14.96	8.15	3.75	40.36	16.02	8.20	4.93	40.36	11.05	6.85	3.75	30.86
cfPWV (m/s)	8.24	1.73	4.42	15.57	8.34	1.75	4.95	15.57	7.90	1.65	4.42	10.69
crPWV (m/s)	10.80	1.35	7.70	13.80	10.68	1.36	7.70	13.80	11.21	1.28	8.70	13.00
PWV Ratio	0.77	0.18	0.37	1.42	0.79	0.19	0.37	1.42	0.70	0.11	0.48	0.96
**Arterial blood pressure**												
aoSBP (mmHg)	111	11	83	131	113	10	87	131	104	10	83	120
aoDBP (mmHg)	75	8	53	94	76	8	53	94	72	8	53	85
baSBP (mmHg)	125	11	102	152	127	11	102	152	119	9	102	138
baDBP (mmHg)	74	7	55	90	75	7	55	90	70	7	56	82

*MV, mean value; SD, standard deviation; Min, Max., minimum and maximum; R, right; L, left; BMI, body mass index; HLD, hyperlipidemic; OM, Omron bioelectrical impedance device; PBF, body fat percentage; PMM, muscle mass percentage; BMR, basal metabolic rate; VFL, visceral fat level (30 levels); PFFM, fat-free mass percentage; FFMI, fat-free mass index; FMI, fat mass index; SBP, DBP, systolic and diastolic blood pressure (suffix: ao: aortic, ba: brachial artery); CCA, CFA, BA, common carotid, common femoral and brachial artery; DD, diastolic diameter. IMT, intima-media thickness; EM, elastic modulus; cfPWV, crPWV, carotid-femoral and carotid-radial pulse wave velocity*.

### Anthropometric and Clinical Evaluation

The participants were asked to avoid exercise, tobacco, alcohol, caffeine, and food intake 4 h before the evaluation, and not to perform strenuous physical activity in the previous 24 h. Additionally, the participants should empty their bladder 30 min before the anthropometric and body composition assessment. A clinical interview and the anthropometric evaluation enabled us to assess CRFs exposure, defined according to the criteria described below (data analysis). A family history of cardiovascular disease was defined by the presence of at least one first-degree (for all the subjects) or second-degree (for subjects ≤ 18 y) relatives with early (<55 y in males; <65 y in females) cardiovascular disease.

**Figure 1:**
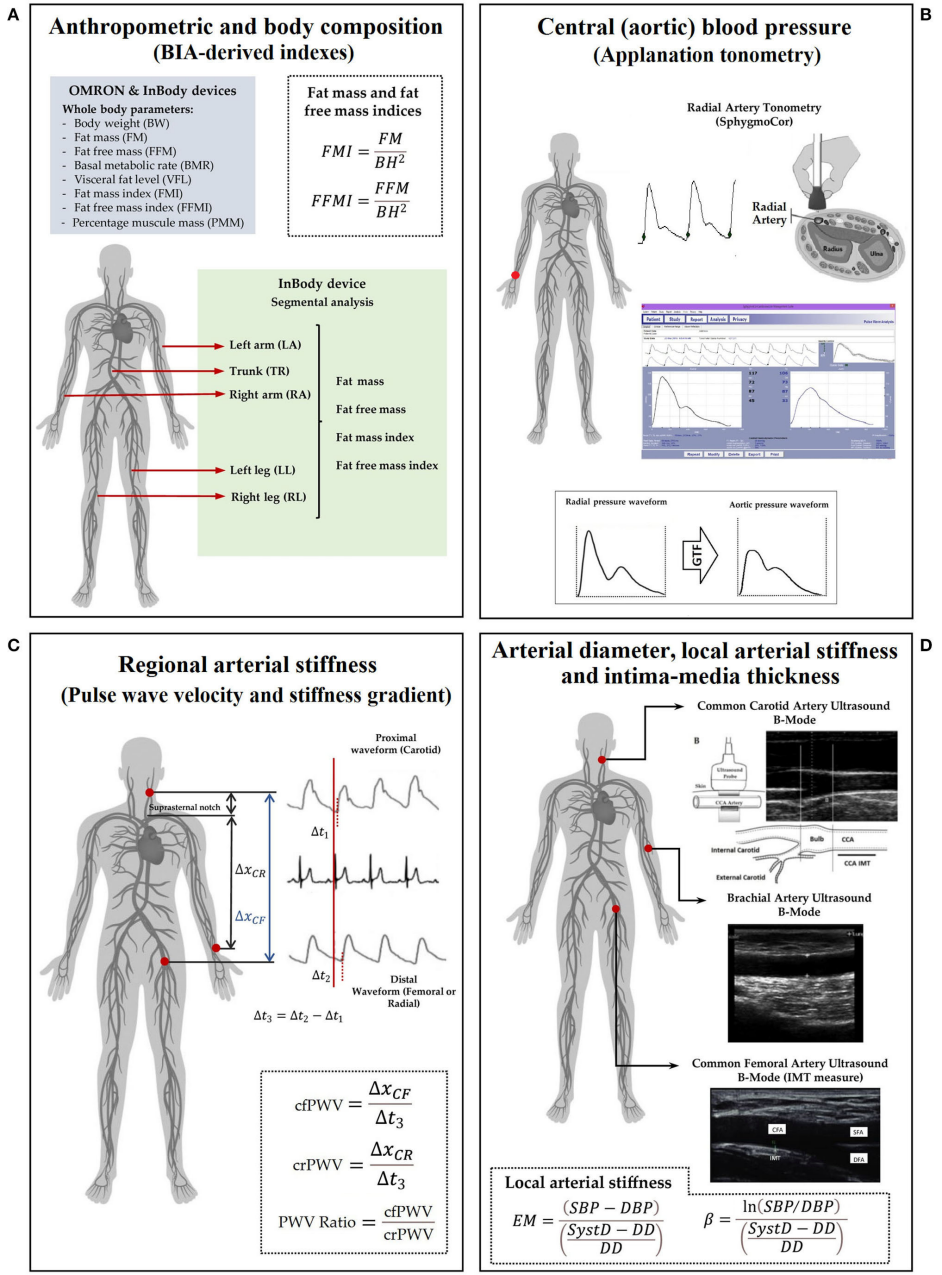
A measurement scheme. Left to right, top to bottom. **(A)** anthropometric evaluation using OMRON HBF-514C (OM) and InBody-120 (IB); **(B)** central and peripheral blood pressure evaluation; **(C)** regional arterial stiffness: PWV (cfPWV and crPWV) and PWV ratio; **(D)** arterial diameter, IMT, and local arterial stiffness. Abbreviations as in text.

Body weight and BH were measured with the participants wearing light clothing and no shoes. BH was measured using a portable stadiometer and recorded to the nearest 0.1 cm. BW, fat mass, fat-free mass, muscle mass, BMR, and visceral fat level (VFL) were measured with two validated BIA devices: (i) mono-frequency (50 kHz) and mono-segmental [Omron HBF-514C (OM), Omron Healthcare, Inc., Illinois, USA)] and (ii) multi-frequency (20 kHz and 100 kHz) multi-segmental [InBody-120 (IB), InBody Co., Seoul, Korea]. To minimize variations due to fluid shifts in the body, the different BIA devices were placed side by side so that the subject could move from unit to unit without wasting time and too much movement. FMI and FFMI were calculated by dividing fat mass and fat-free mass by the square of the BH, respectively. Specific variables per segment (i.e., FMI and FFMI of trunk, upper, and lower limbs) were also calculated using the fat mass and fat-free mass of each segment and the same BH. BMI was calculated as BW divided by the square of BH. Body fat percentage (PBF) was calculated, dividing body fat mass by BW and multiplied by 100 (Figure 1). Detailed information on the validity of BIA measurements using InBody and OMRON technology, and technical characteristics of both devices can be found in Supplementary File 1.

### Cardiovascular Evaluation

All measurements were performed in a temperature-controlled environment (21–23°C), with the subject in supine position and after resting for at least 10–15 min. Cardiovascular evaluation in the CUiiDARTE project included assessing hemodynamic, structural, and functional parameters (21–27). In this study, we focused on central and peripheral BP levels, a beat-to-beat arterial diameter, intima-media thickness (IMT), and regional and local arterial stiffness indexes.

#### Peripheral and Central Blood Pressure

Using a validated oscillometric device (HEM-433INT; Omron Healthcare Inc., Lake Forest, IL, USA), heart rate and brachial systolic and diastolic BP (baSBP, baDBP) were recorded simultaneously and/or immediately before or after each non-invasive echographic, tonometric, and oscillometric record. Brachial mean BP (baMBP) was quantified as baDBP + (baSBP-baDBP)/3.

Systolic and diastolic aortic BPs (aoSBP, aoDBP) were non-invasively obtained by means of applanation tonometry [SphygmoCor-CvMS (SCOR), v.9, AtCor-Medical, Australia] (20, 22). Briefly, radial BP waveform was obtained by tonometry, and the aortic BP (aoBP) waveform was then derived indirectly from the calibration of the acquired radial waveforms and application of a general transfer function. Radial waveforms were calibrated with baDBP and baMBP (Figure 1).

#### Regional Arterial Stiffness and Central-to-Peripheral Stiffness Gradient

Carotid-femoral (cfPWV, a marker of aortic stiffness) and carotid-radial pulse wave velocity (crPWV, a marker of upper arm arteries stiffness) were obtained by tonometry (SCOR) (23, 28). cfPWV and crPWV were obtained as the median of three recordings. The pulse wave velocity (PWV) ratio (a marker of a central-peripheral stiffness gradient) was quantified: cfPWV/crPWV (23, 29, 30) (Figure 1).

#### Local Arterial Stiffness, Diameter, and Intima-Media Thickness

Left (L-) and right (R-) common carotid arteries (CCA), common femoral artery (CFA), and left brachial artery (BA) were analyzed using ultrasound (6–13 MHz, M-Turbo, Sonosite Inc., WA, USA). Sequences of images (30 s, B-Mode, longitudinal views) were stored for off-line analysis. A beat-to-beat diameter and IMT waves were obtained using border detection software (HemoDyn 4-M, Dinap s.r.l., Bs.As., Argentina). Peak systolic (SysD) and end-diastolic (DD) diameters and IMT (far wall, end diastole) values were obtained by averaging at least 20 beats. The CCA diameter and IMT were measured a centimeter proximal to the carotid bulb. The CFA diameter was measured in the penultimate centimeter proximal to the bifurcation. BA measurements were acquired at the elbow level in a straight segment of at least one-centimeter long (26) (Figure 1).

Local arterial stiffness was quantified by the elastic modulus (EM) and the beta index (β). The EM measures the ability of the artery to change its dimensions in response to the BP caused by cardiac ejection [BP change required for (theoretic) 100% increase in diameter]: EM = (SBP-DBP)/(SysD-DD)/DD). To minimize the impact that BP levels have on stiffness, the β was quantified: β = Ln (SBP/DBP)/[(SysD-DD)/DD]. The baSBP and baDBP were used to quantify CFA and BA EMs and βs; aoSBP and aoDBP were used to quantify CCA EM and β (Figure 1).

### Data Analysis

#### Standardized Cardiovascular Variables (z-Scores)

Considering specific inclusion and exclusion criteria, the subjects to be included in the reference group were identified to get standardized cardiovascular variables expressed as z-scores (Supplementary File 2: Table S1). As in previous works, the reference group was determined by selecting a healthy sub-population from the CUiiDARTE database (*n* = 1,688) that included children, adolescents, and adults who did not meet any of the following exclusion criteria: (i) history of cardiovascular disease; (ii) use of BP-, lipid- or glucose-lowering drugs; (iii) arterial hypertension (≥18 y: baSBP ≥140 mmHg or baDBP ≥90 mmHg; <18 y: baSBP and baDBP >95th percentile for sex, age, and BH); (iv) current smoking; (v) diabetes, defined as self-reported or fasting plasma glucose ≥126 mg/dL (if available); (vi) dyslipidemia, defined as self-reported or total cholesterol ≥ 240 mg/dL or HDL cholesterol <40 mg/dL (if available); (vii) obesity (≥18 y: BMI ≥30 kg/m2; <18 y: z-BMI ≥2.0) (23–25). None of the subjects had congenital or chronic conditions, infectious diseases, or significant cardiac arrhythmias.

Once the reference group was built, age-related equations were obtained for mean value (MV) and standard deviation (SD). To this end, we implemented parametric regression methods based on various types of models (fractional polynomials, polynomial, ratios of polynomials) (23–25, 31, 32).

Figure S1, in Supplementary File 3, exemplifies (for a baSBP variable) the fractional polynomial models used to obtain these equations. This procedure provides different age-related equations for each model to calculate z-scores, and then the most adjusted model is chosen to calculate an individual's cardiovascular z-scores (Supplementary File 2: Table S2). Subsequently, by using these equations, we were able to quantify the z-score levels of each arterial variable in the subjects who had BIA-derived measurements (Supplementary File 2: Tables S3, S6; Supplementary File 3: Figure S1).

#### Mono-Segmental BIA-Derived Body Composition Indexes: Correlation and Regression Models

**Figure 2:**
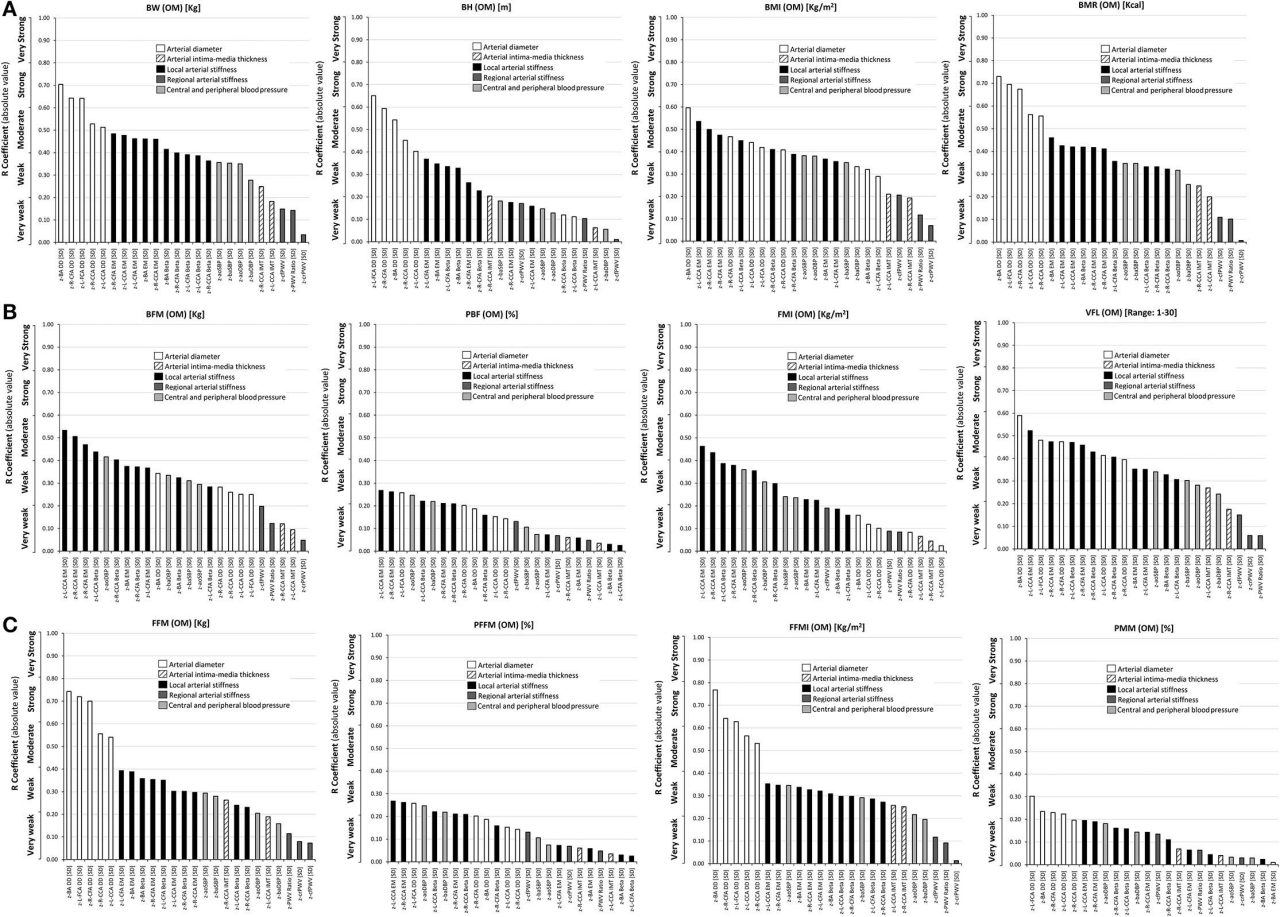
Simple (Pearson, r) correlation coefficients (absolute value), ranked from highest to lowest r value, for: **(A)** Top: classical anthropometric indexes (BW, BH, BMI, BMR), **(B)** Middle: body fat mass indexes (BFM, PFM, FMI) and VFL, and **(C)** Bottom: fat-free mass indexes (FFM, PFFM, FFMI, PMM), and cardiovascular z-scores. Variables were obtained with the Omron BIA device (OM). Abbreviations as in text.

**Figure 3:**
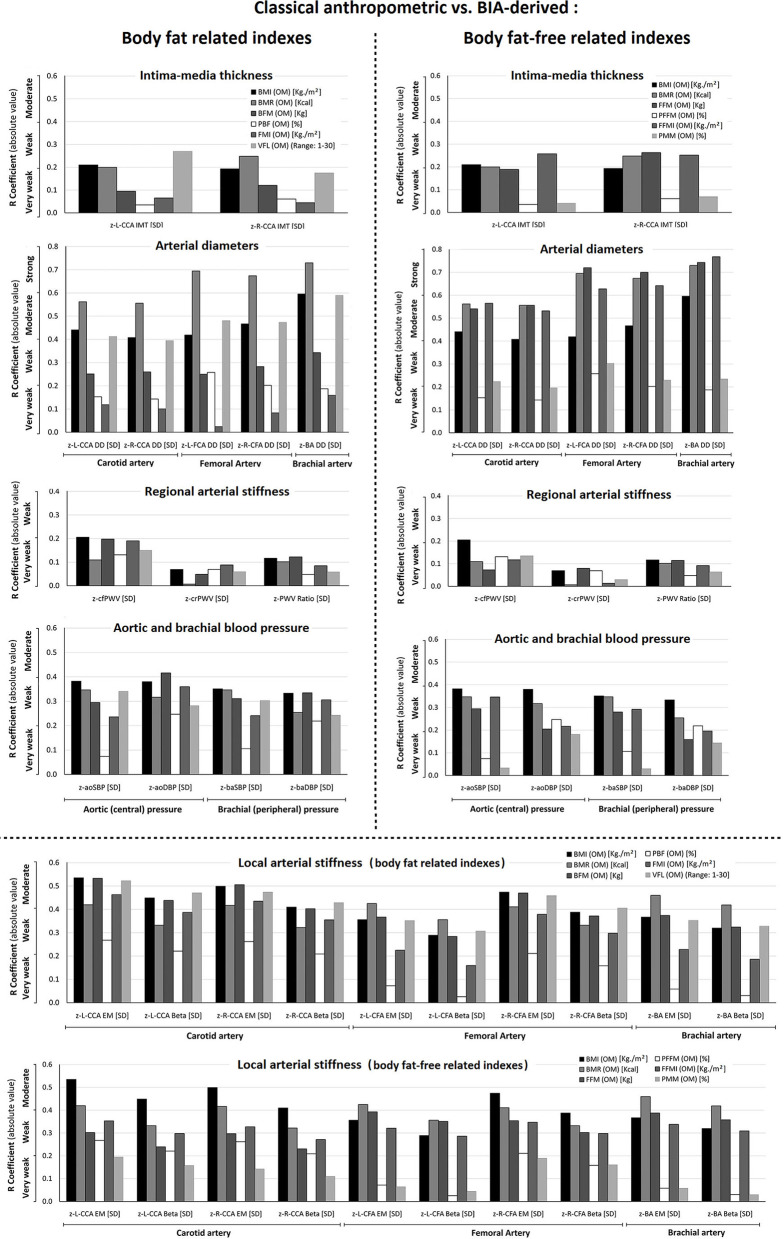
Comparison of the levels of association (r, absolute value) between cardiovascular z-scores and classical anthropometric (BMI, BMR) vs. fat mass indexes (BFM, PBF, FMI, VFL), and classical anthropometric (BMI, BMR) vs. fat-free mass indexes (FFM, PFFM, FFMI, PMM). The variables were obtained with the Omron BIA device (OM). Abbreviations as in text.

**Figure 4:**
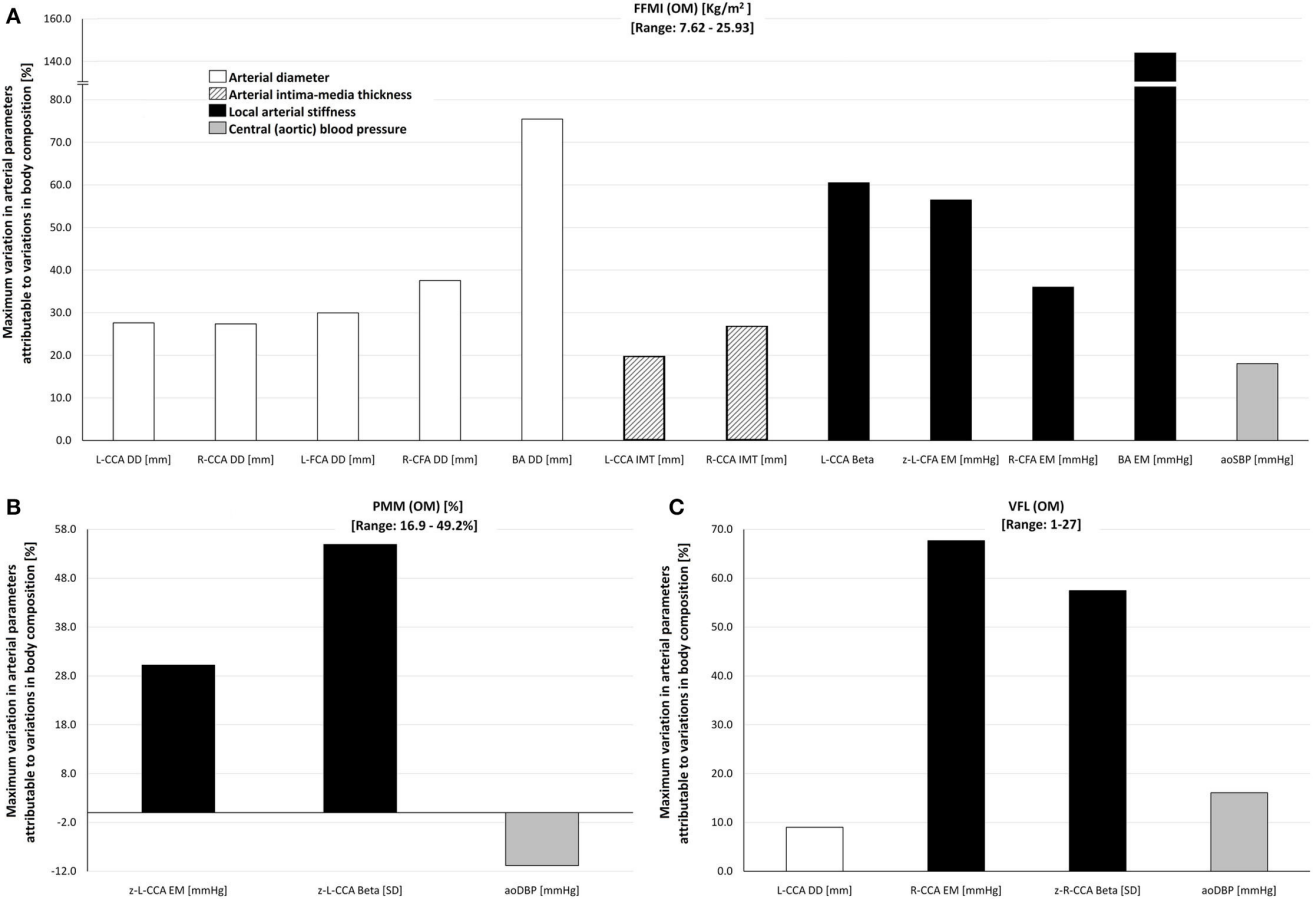
Maximum percentage of variation of cardiovascular variables attributable to inter-individual variations in **(A)** FFMI (range: 7.62–25.95 kg/m^2^), **(B)** PMM (range: 16.9–49.2%), and **(C)** VFL (range: 1–27) in the study group (*n* = 538), obtained by the Omron BIA device (OM). For detailed information, see Table 3. Abbreviations as in text.

Two-tailed simple bivariate correlations were performed to quantify the strength of association between exposure to CRFs and classical anthropometric and mono-segmental BIA-derived indexes, and cardiovascular z-scores (Figures 2, 3; Supplementary File 2: Table S4). Multiple linear regression models (Input: stepwise) were constructed, considering the cardiovascular z-scores as dependent variables and CRFs, classical anthropometric and mono-segmental BIA-derived indexes as independent variables (Table 2). In addition, by using: (i) multiple linear regression-derived non-standardized B coefficients, (ii) MV and SD data (the reference group), and (iii) the minimum and maximum values (range) of each mono-segmental BIA-derived indexes, it was possible to quantify for each arterial variable (in the respective units): (i) the maximum variation that could be associated (attributed) to the different values obtained on body composition indexes and (ii) the variations that could be (theoretically) expected, considering the inter-individual variations on BIA-derived body composition indexes (Table 3, Figure 4).

**Table 2:** Association between cardiovascular z-scores (dependent variable) and cardiovascular risk factors, classical anthropometric and body composition indices (independent variables) (OMRON HBF-514C device).

**Dependent variable**	**Independent variables**	**Bu**	**SE**	**95% CILL**	**95% CIUL**	**Bs**	***p***	**VIF**	***R***	***R*** **^2^**	***Adj R*** **^2^**
**Arterial structural parameters**											
z-L-CCA DD (SD)	Constant	−2.331	0.397	−3.112	−1.550		<0.001		0.51	0.26	0.25
	FFMI (OM) (kg/m^2^)	0.159	0.025	0.109	0.209	0.422	<0.001	2.16			
	Age (years)	−0.014	0.003	−0.021	−0.008	−0.265	<0.001	1.91			
	Diabetes	0.879	0.299	0.292	1.467	0.140	0.003	1.08			
	VFL (OM)	0.037	0.017	0.003	0.071	0.174	0.035	3.23			
z-R-CCA DD (SD)	Constant	−2.890	0.340	−3.558	−2.221		<0.001		0.43	0.18	0.18
	FFMI (OM) (kg/m^2^)	0.172	0.019	0.135	0.210	0.435	<0.001	1.00			
z-L-FCA DD (SD)	Constant	−1.217	0.604	−2.407	−0.027		0.045		0.69	0.48	0.47
	Sex (1:Female; 0:Male)	−0.863	0.171	−1.201	−0.525	−0.443	<0.001	3.54			
	History of CVD	−0.586	0.265	−1.108	−0.064	−0.108	0.028	1.09			
	FFMI (OM) (kg/m^2^)	0.108	0.032	0.045	0.171	0.318	0.001	4.11			
	Age (years)	−0.006	0.003	−0.012	−0.001	−0.130	0.021	1.44			
z-R-CFA DD (SD)	Constant	−2.110	0.647	−3.384	−0.836		0.001		0.66	0.44	0.44
	FFMI (OM) (kg/m^2^)	0.141	0.032	0.078	0.204	0.374	<0.001	3.12			
	Sex (1:Female; 0:Male)	−0.695	0.182	−1.053	−0.337	−0.325	<0.001	3.12			
z-BA DD (SD)	Constant	−4.720	0.373	−5.457	−3.984		<0.001		0.72	0.52	0.52
	FFMI (OM) (kg/m^2^)	0.258	0.020	0.219	0.296	0.727	<0.001	1.00			
z-L-CCA IMT (SD)	Constant	−1.002	0.361	−1.711	−0.292		0.006		0.22	0.05	0.04
	FFMI (OM) (kg/m^2^)	0.069	0.020	0.029	0.109	0.181	0.001	1.04			
	Diabetes	0.678	0.337	0.016	1.340	0.107	0.045	1.04			
z-R-CCA IMT (SD)	Constant	−1.565	0.431	−2.413	−0.717		<0.001		0.34	0.12	0.11
	FFMI (OM) (kg/m^2^)	0.102	0.024	0.055	0.150	0.217	<0.001	1.04			
	Diabetes	1.395	0.400	0.608	2.182	0.179	0.001	1.04			
	Family History CVD	0.689	0.223	0.250	1.129	0.155	0.002	1.00			
**Arterial functional parameters**											
z-L-CCA EM (SD)	Constant	−2.995	0.422	−3.824	−2.165		<0.001		0.39	0.15	0.14
	BMI (OM) (kg/m^2^)	0.086	0.011	0.064	0.107	0.412	<0.001	1.13			
	PMM (OM) (%)	0.029	0.007	0.014	0.043	0.204	<0.001	1.13			
z-L-CCA Beta (SD)	Constant	−2.505	0.419	−3.329	−1.680		<0.001		0.3	0.10	0.09
	FMI (OM) (kg/m^2^)	0.105	0.018	0.069	0.141	0.430	<0.001	2.18			
	PMM (OM) (%)	0.054	0.009	0.035	0.072	0.425	<0.001	2.18			
z-R-CCA EM (SD)	Constant	−0.917	0.148	−1.208	−0.626		<0.001		0.50	0.25	0.24
	VFL (OM)	0.086	0.013	0.060	0.113	0.422	<0.001	1.02			
	Diabetes	0.847	0.311	0.232	1.461	0.177	0.007	1.03			
	Family History CVD	0.477	0.203	0.076	0.878	0.151	0.020	1.00			
z-R-CCA Beta (SD)	Constant	−0.929	0.148	−1.221	−0.637		<0.001		0.43	0.18	0.17
	VFL (OM)	0.079	0.013	0.053	0.105	0.404	<0.001	1.00			
	Family History CVD	0.462	0.204	0.060	0.864	0.152	0.025	1.00			
z-L-CFA EM (SD)	Constant	−0.942	0.424	−1.777	−0.106		0.027		0.18	0.03	0.03
	FFMI (OM) (kg/m^2^)	0.067	0.023	0.021	0.113	0.184	0.004	1.00			
z-L-CFA Beta (SD)	No variables were entered into the equation.										
z-R-CFA EM (SD)	Constant	−0.697	0.448	−1.579	0.185		0.121		0.23	0.05	0.04
	Hypertension	0.405	0.175	0.060	0.751	0.155	0.022	1.14			
	FFMI (OM) (kg/m^2^)	0.050	0.025	0.000	0.099	0.133	0.050	1.14			
z-R-CFA Beta (SD)	Constant	0.158	0.069	0.023	0.293		0.022		0.14	0.02	0.01
	Hypertension	0.332	0.151	0.034	0.630	0.141	0.029	1.00			
z-BA EM (SD)	Constant	−1.898	0.835	−3.552	−0.244		0.025		0.36	0.13	0.11
	FFMI (OM) (kg/m^2^)	0.153	0.041	0.072	0.234	0.330	<0.001	1.03			
	Age (years)	−0.019	0.008	−0.035	−0.003	−0.212	0.018	1.03			
z-BA Beta (SD)	Constant	−2.068	0.728	−3.510	−0.626		0.005		0.24	0.06	0.05
	FFMI (OM) (kg/m^2^)	0.104	0.037	0.030	0.178	0.249	0.006	1.00			
z-cfPWV (SD)	Constant	0.056	0.063	−0.067	0.180		0.370		0.24	0.06	0.05
	Diabetes	1.097	0.341	0.427	1.767	0.168	0.001	1.03			
	Hypertension	0.430	0.156	0.123	0.738	0.144	0.006	1.03			
z-crPWV (SD)	No variables were entered into the equation										
z-PWV Ratio (SD)	No variables were entered into the equation										
**Arterial Blood Pressure**											
z-aoSBP (SD)	Constant	−2.444	0.351	−3.135	−1.753		<0.001		0.37	0.14	0.13
	FFMI (OM) (kg/m^2^)	0.107	0.025	0.058	0.155	0.273	<0.001	1.65			
	BMI (OM) (kg/m^2^)	0.031	0.014	0.004	0.059	0.140	0.027	1.65			
z-aoDBP (SD)	Constant	0.579	0.251	0.085	1.073		0.022		0.47	0.22	0.21
	VFL (OM)	0.055	0.011	0.033	0.077	0.261	<0.001	1.29			
	PMM (OM) (%)	−0.030	0.007	−0.043	−0.016	−0.202	<0.001	1.02			
	Diabetes	0.993	0.295	0.413	1.573	0.163	0.001	1.07			
	History of CVD	−0.845	0.331	−1.496	−0.195	−0.126	0.011	1.11			
	Hypertension	0.323	0.152	0.024	0.622	0.116	0.035	1.35			
z-baSBP (SD)	Constant	−2.456	0.584	−3.609	−1.302		<0.001		0.37	0.13	0.13
	BMI (OM) (kg/m^2^)	0.103	0.021	0.061	0.144	0.373	<0.001	1.00			
z-baDBP (SD)	Constant	−2.036	0.591	−3.204	−0.867		0.001		0.32	0.10	0.10
	BMI (OM) (kg/m^2^)	0.090	0.021	0.048	0.132	0.328	<0.001	1.00			

*Bu y Bs: un- and standardized coefficients. R, Pearson coefficient; R^2^, adjusted squared R; VIF, variance inflation factor (a VIF <5 was defined to evaluate (discard) multicollinearity). SE, standard error; LL, UL, lower and upper limits; CI, confidence interval; z: z-score. BW and BH, bodyweight and height; BMI, body mass index; OM, Omron bioelectrical impedance analysis device; PBF, body fat percentage; PMM, muscle mass percentage; VFL, visceral fat level; PFFM, fat-free mass percentage; FFMI, fat-free mass index; FMI, fat mass index; R, right. L, left; SBP, DBP, systolic and diastolic pressure (suffix: ao: aortic, ba: brachial artery). CCA, CFA, BA; common carotid, common femoral, and brachial artery; DD, diastolic diameter; IMT, intima-media thickness; EM, elastic modulus; cfPWV, crPWV, carotid-femoral and carotid-radial pulse wave velocity; PWV ratio, cfPWV/crPWV ratio; SD, standard deviation; CVD, cardiovascular disease. All anthropometric variables were included in the multiple linear regression. For diabetes, history of CVD, family history of CVD, hypertension: 1: Yes and 0: No. Only significant (p < 0.05) independent variables entered in the models (Stepwise) are shown*.

**Table 3:** Impact of interindividual variations of body composition indices (independent variables) on cardiovascular properties (dependent variables) (OMRON HBF-514C device).

**Dependent variable**					**Cardiovascular differences attributable to FFMI variations**					
**Cardiovascular variable**	**Age (y)**	**MV (RG)**	**SD RG)**	**Bu**	**5 units**	**10 units**	**15 units**	**20 units**	**Δ (Max-Min)**	**Δ%**
**Fat-free Mass Index (FFMI) (kg/m2)[MV: 17.51; SD: 2.72; Range: 7.62 - 25.93]**										
L-CCA DD (mm)	10	4.71	0.52	0.16	0.41	0.83	1.24	1.65	1.51	32.05
	30	6.34	0.50		0.40	0.80	1.19	1.59	1.46	22.98
	50	6.72	0.66		0.52	1.04	1.57	2.09	1.91	28.42
	70	7.04	0.65		0.52	1.04	1.56	2.08	1.90	27.07
R-CCA DD (mm)	10	5.77	0.50	0.17	0.43	0.86	1.29	1.72	1.58	27.31
	30	6.45	0.51		0.44	0.88	1.32	1.76	1.61	24.94
	50	6.81	0.64		0.55	1.11	1.66	2.22	2.03	29.80
	70	7.19	0.63		0.54	1.08	1.62	2.15	1.97	27.43
L-FCA DD (mm)	10	5.51	0.68	0.11	0.37	0.73	1.10	1.47	1.34	24.42
	30	7.75	1.13		0.61	1.22	1.83	2.44	2.23	28.82
	50	8.33	1.47		0.79	1.59	2.38	3.18	2.91	34.92
	70	8.59	1.38		0.74	1.49	2.23	2.98	2.72	31.70
R-CFA DD (mm)	10	5.53	0.68	0.14	0.48	0.96	1.45	1.93	1.76	31.87
	30	7.77	1.07		0.75	1.50	2.25	3.00	2.74	35.30
	50	8.32	1.34		0.94	1.88	2.82	3.76	3.44	41.37
	70	8.33	1.35		0.95	1.90	2.85	3.80	3.48	41.74
BA DD (mm)	10	2.71	0.35	0.26	0.45	0.90	1.35	1.80	1.65	60.77
	30	3.70	0.62		0.80	1.60	2.40	3.20	2.93	79.08
	50	4.08	0.78		1.01	2.02	3.03	4.03	3.69	90.39
	70	4.21	0.64		0.82	1.65	2.47	3.30	3.02	71.61
L-CCA IMT (mm)	10	0.43	0.05	0.07	0.02	0.03	0.05	0.07	0.06	14.2
	30	0.55	0.08		0.03	0.06	0.09	0.12	0.11	19.3
	50	0.69	0.10		0.03	0.07	0.10	0.13	0.12	17.8
	70	0.85	0.19		0.06	0.13	0.19	0.26	0.24	27.8
R-CCA IMT (mm)	10	0.44	0.04	0.10	0.02	0.04	0.06	0.08	0.07	16.69
	30	0.54	0.10		0.05	0.10	0.15	0.19	0.18	32.74
	50	0.67	0.08		0.04	0.09	0.13	0.17	0.16	23.67
	70	0.83	0.15		0.08	0.16	0.23	0.31	0.29	34.24
L-CCA Beta	10	4.71	1.83	0.10	0.96	1.92	2.88	3.84	3.51	74.49
	30	7.19	1.90		0.99	1.99	2.98	3.98	3.64	50.64
	50	9.57	2.59		1.36	2.71	4.07	5.42	4.96	51.85
	70	11.79	4.01		2.10	4.20	6.30	8.40	7.68	65.14
z-L-CFA EM (mmHg)	10	829	339	0.07	114	227	341	454	416	50.14
	30	1,235	617		207	413	620	826	756	61.22
	50	1,243	579		194	388	581	775	709	57.09
	70	1,128	529		177	354	531	708	648	57.43
R-CFA EM (mmHg)	10	823	315	0.05	78	156	234	313	286	34
	30	1,185	520		129	258	387	516	472	39
	50	1,116	453		112	225	337	450	411	36
	70	919	329		81	163	245	326	298	32
BA EM (mmHg)	10	943	502	0.15	385	770	1,154	1,539	1,384	147
	30	1,265	690		529	1,059	1,560	2,080	1,904	151
	50	1,385	696		534	1,067	15,73	2,097	1,919	139
	70	1,475	733		562	1,125	1,657	2,210	2,022	137
aoSBP (mmHg)	10	91.7	8.31	0.11	4.4	8.8	13.2	17.7	16.2	17.6
	30	106.1	10.1		5.3	10.7	16.0	21.4	19.6	18.4
	50	109.6	9.3		4.9	9.9	14.8	19.8	18.1	16.5
	70	111.1	11.1		5.9	11.8	17.8	23.7	21.7	19.5
**Muscle Mass Percentage (PMM) (%)(MV: 32.32; SD: 7.12; Range: 16.9 - 49.2%)**										
z-L-CCA EM (mmHg)	10	386	155	0.03	22	44	67	89	143	37
	30	676	179		26	52	77	103	166	25
	50	935	272		39	78	117	156	252	27
	70	1,143	395		57	113	170	227	366	32
z-L-CCA beta (SD)	10	4.71	1.83	0.05	0.49	0.99	1.48	1.97	3.18	68
	30	7.19	1.90		0.51	1.02	1.53	2.04	3.30	46
	50	9.57	2.59		0.70	1.39	2.09	2.79	4.50	47
	70	11.79	4.01		1.08	2.16	3.24	4.31	6.97	59
aoDBP (mmHg)	10	62.1	8.2	−0.03	−1.2	−2.4	–.6	−4.8	−7.8	−12.7
	30	71.1	8.7		−1.2	−2.5	−3.8	−5.1	−8.3	−11.7
	50	75.0	7.5		−1.1	−2.2	−3.3	−4.4	−7.2	−9.6
	70	73.9	7.2		−1.0	−2.1	−3.2	−4.2	−6.9	−9.4
**Dependent variable**					**Cardiovascular differences attributable to FFMI variations**					
**Cardiovascular Variable**	**Age (y)**	**MV (RG)**	**SD RG)**	**Bu**	**5 units**	**10 units**	**15 units**	**20 units**	**Δ (Max-Min)**	**Δ%**
**Visceral Fat Level (VFL) (MV: 7.18; SD: 4.94; Range: 1-27)**										
L-CCA DD (mm)	10	4.71	0.52	0.04	0.09	0.19	0.28	0.38	0.49	10.46
	30	6.34	0.50		0.09	0.18	0.27	0.37	0.48	7.50
	50	6.72	0.66		0.12	0.24	0.36	0.48	0.62	9.27
	70	7.04	0.65		0.12	0.24	0.36	0.48	0.62	8.83
R-CCA EM (mmHg)	10	399	121	0.09	52	104	157	209	271	68
	30	661	166		72	143	215	286	372	56
	50	875	267		115	231	346	461	599	69
	70	1,060	368		159	317	476	635	825	78
z-R-CCA Beta	10	4.91	1.39	0.08	0.55	1.11	1.66	2.21	2.88	58.59
	30	7.06	1.77		0.70	1.40	2.10	2.81	3.65	51.68
	50	9.01	2.62		1.04	2.08	3.12	4.16	5.41	60.04
	70	10.93	3.15		1.25	2.50	3.75	5.00	6.50	59.51
aoDBP (mmHg)	10	62.2	8.2	0.05	2.2	4.5	6.7	9.0	11.7	18.8
	30	71.1	8.7		2.4	4.8	7.1	9.5	12.4	17.4
	50	75.1	7.5		2.1	4.1	6.2	8.2	10.7	14.3
	70	74.0	7.2		2.0	4.0	5.9	7.9	10.3	13.9

RG, reference group; MV, mean value; SD, standard deviation; CV, cardiovascular; Bu, beta un-standardized; Min, Max, minimum and maximum; R, right; L, left; SBP, DBP, systolic and diastolic pressure (suffix: ao: aortic, ba: brachial artery); CCA, CFA, BA, common carotid, common femoral, and brachial artery. DD; diastolic diameter. IMT, intima-media thickness;. EM, elastic modulus. “Δ (Max-Min)” is expressed as absolute value. Δ% was quantified as: [(Max-Min)/MV]^*^100.

#### Agreement Between Mono-and Multi-Segmental BIA Devices

Lin's concordance correlation coefficient and Bland-Altman tests were performed to evaluate the agreement between BIA devices (Supplementary File 2: Table S7; Supplementary File 3: Figure S2). Bland-Altman plots correspond to the mean of the methods considered (x-axis) against their difference (y-axis). Systematic error (bias) was considered present if mean error was significantly different from 0, whereas proportional error was considered present if the slope of the linear regression was statistically significant. Descriptive statistics obtained for the participants evaluated with multi-frequency BIA device (InBody-120) is shown in Supplementary File 2: Table S5.

#### Multi-Segmental BIA-Derived Body Composition Indexes: Correlation and Regression Models

Finally, using the information from the multi-segmental BIA device, an analysis similar to the one previously reported was performed. Correlation analyses were implemented to quantify the association between CRFs, classical anthropometric indexes, and multi-segmental BIA-derived indexes obtained for “total body,” “trunk,” “upper limb,” and “lower limb” segments and cardiovascular z-scores (Supplementary File 2: Table S8; Supplementary File 3: Figures S3–S8).

### Statistical Analysis

According to the central limit theorem, a normal distribution was considered (taking into account Kurtosis and Skewness coefficients distribution and number of studied subjects; sample size >30) (33). The number of the subjects included was much higher than the minimum required sample size, both to construct the reference group to obtain the MV and SD equations (included: 1,688, minimum required sample size: 377), and to perform the agreement and/or association analyses (included: 538 for OM and 110 for IB, a minimum required sample size: 103). Consequently, the number of the subjects studied was higher than the minimum number calculated for: α = 0.05, β = 0.20, anticipated effect size = 0.15 (medium), and a total number of predictors in the multiple linear regression model = 7.

Even in this conservative context, when making associations, we performed Bootstrapping of the samples as a strategy to evaluate whether potential associations observed between cardiovascular z-scores and body composition indexes do maintain even after analyzing different random sampling settings (resampling with replacement from the original sample). In other words, with this mechanism, any initial *p* < 0.05 may no longer be significant after the “fictional random re-sampling” (i.e., bootstrapping). This type of test obligates the investigators to consider only those significant p values that replicate in both statistical scenarios (the actual sample and bootstrapping sampling). To this end, Bootstrap-derived 95% confidence intervals (1,000 samples) were obtained, applying bias-corrected and accelerated methods for computing confidence interval limits [lower and upper limits (LL and UL, respectively)]. The association was considered significant only if the 95% confidence interval of Pearson's coefficient, quantified by bootstrapping, did not contain the 0 value.

Evans's empirical classifications of interpreting correlation strength by using r were applied: *r* < 0.20, very weak; *r*: 0.20–0.39, weak; *r*: 0.40–0.59, moderate; *r*: 0.60–0.79, strong; *r* ≥ 0.80, very strong (34). Analyses were done using SPSS (IBM-SPSS Inc., Chicago, IL, USA), MedCalc (MedCalc Inc., Ostend, Belgium) and NCSS 2020 (NCSS, Kaysville, UT; www.NCSS.com) software. A *p* < 0.05 was considered statistically significant.

## Results

The subjects' characteristics evaluated with mono-segmental BIA are shown in Table 1. There was a balanced distribution of sex (47% female), and a wide age range (7–85 years). The levels of CRFs exposure and drug use were similar to general population. There was a wide range of variation in fat mass and fat-free mass indexes. Inter-individual variations in fat mass indexes were for PBF (5.4–68.0%), FMI (0.9–28.6 kg/m^2^), VFL (1–27, scale between 1 and 30 levels), while, in fat-free mass, indexes were for PMM% (16.9–49.2%), FFMI (7.6–25.9 kg/m^2^), and PFFM (32.−94.6%).

Additionally, there was a wide inter-individual variation in arterial properties. For instance, while certain subjects presented z-scores lower than −2, others had values higher than +2, or even than +4 (Supplementary File 2: Tables S3, S6; Supplementary File 3: Figure S1).

### Classical Anthropometric, Body and Visceral Fat, and Fat-Free Mass Indexes (Mono-Segmental BIA-Derived): Association With Arterial Variations (Aim 1)

When comparing classical indexes (BW, BH, BMI, and BMR), those that showed the greatest association with cardiovascular z-scores varied, depending on the arterial parameter and segment, although BH never showed the greatest association with any of the arterial parameters. For BMR, the highest levels of association were obtained for CCA and CFA z-diameters (*r*: 0.56–0.73), z-R-CCA IMT (*r*: 0.25) and BA stiffness (*r*: 0.42–0.46). BMI showed the highest levels of association with z-L-IMT (*r*: 0.25), CCA stiffness (*r*: 0.41–0.55), and z-cfPWV (*r*: 0.21), whereas BW showed the highest level of association with CFA and BA z-stiffness (*r*: 0.39–0.48), the z-PWV ratio (*r*: 0.14), and z-baSBP (*r*: 0.35). Therefore, except for BH, classical indexes had heterogeneous levels of association with arterial z-scores, but never these correlations reached strong association values (Figure 2, Supplementary File 2: Table S4).

Body weight, BMR, and BH showed the highest levels of association with z-diameters, followed by z-local stiffness. BMI showed a heterogeneous distribution, showing different levels of association between z-diameters and z-local stiffness. Regarding regional z-stiffness, the four indexes showed a “very weak” association (the lowest levels observed). Regardless of the classical index evaluated, from highest to lowest, a hierarchical order in the levels of association was observed: diameters or local stiffness > BP (aortic, brachial) > regional stiffness (Figure 2, Supplementary File 2: Table S4).

In general, higher mono-segmental BIA-derived fat mass indexes (i.e., BFM, PBF, FMI, VFL) were associated with (i) higher z-carotid, femoral and brachial local stiffness, and (ii) higher z-aoBP and z-baBP. BFM, FMI, and mainly VFL showed “moderate” levels of associations (*r*: 0.40–0.60) with cardiovascular z-scores, whereas the lowest levels were obtained for PBF (*r* < 0.3). Furthermore, z-arterial diameters and z-BP were mainly associated with VFL, FMI, and BFM, respectively. VFL showed the greatest value of association with cardiovascular z-scores (Figure 2, Supplementary File 2: Table S4).

Considering each index individually, both BFM and FMI showed the highest levels of association with z-local stiffness, and only weak associations with z-diameters, whereas the VFL showed strong associations with both z-local stiffness and z-diameters (Figure 2, Supplementary File 2: Table S4). The levels of association with z-regional stiffness were very low. Additionally, considering the body fat mass indexes (BFM and FMI, except for visceral fat), there was the following hierarchical order in associations: local arterial stiffness > BP (aortic, brachial) > arterial diameters > CCA IMT and regional arterial stiffness (Figure 2). When considering visceral fat, the following order was shown: diameters and local stiffness > BP > CCA IMT and regional stiffness.

Higher fat-free mass indexes (especially FFM and FFMI) were associated with higher: (i) CCA, CFA, and BA z-diameter, (ii) CCA, CFA, and BA z-stiffness, and (iii) z-BP (aortic, brachial). Like fat mass indexes, the fat-free indexes showed a “very weak” level of association with z-regional stiffness. Both PFFM and PMM reported the lowest levels of association with cardiovascular z-scores (*r* < 0.4). The FFMI, followed by FFM, showed the highest number of associations with cardiovascular z-scores. Both indexes showed (i) “moderate” and “strong” association with z-diameters, followed by (ii) “weak or moderate” association with z-local stiffness. Unlike fat mass indexes, FFM and FFMI showed a marked difference between the levels of association with z-diameters and z-local stiffness (Figure 2, Supplementary File 2: Table S4).

The joint association analysis of fat (BFM, FMI, VFL) and fat-free mass indexes (FFM and FFMI) with cardiovascular z-scores showed that all significant associations were positive; besides, a higher index was associated with a higher cardiovascular z-score (Supplementary File 2: Table S4).

### Comparative Analysis of Classical, Body, and Visceral Fat, and Fat-Free Mass Indexes (Mono-Segmental BIA-Derived) as Explanatory Variables of Arterial Variations (Aim 2)

#### Bivariate Analysis

The analysis of z-local stiffness was characterized by relatively strong associations with BMI and BMR, in which neither of the indexes of fat mass (especially VFL) nor fat-free mass exceeded these levels of association. Regarding regional stiffness, the associations with fat and fat-free mass indexes were comparatively “very weak,” being these values lower than BMI and BMR (Figure 3, Supplementary File 2: Table S4).

Association analyses between z-structural and fat-free mass indexes suggest that FFM and FFMI showed at least a stronger association than BMI and BMR. Conversely, although VFL showed the highest “*r*” value (a moderate level), BMI and BMR showed stronger associations with z-diameters (Figure 3, Supplementary File 2: Table S4). The associations between fat and fat-free mass indexes and z-IMT were very weak. Same weak associations were observed when considering BMI and BMR.

We found less or similar levels of association between fat and fat-free mass indexes (compared to BMI and BMR) with respect to z-BP (aortic, brachial) (Figure 3, Supplementary File 2: Table S4).

#### Multivariate Analyses

In general terms, regardless of age, sex, CRFs, and classical anthropometric indexes, variations in cardiovascular z-scores can be explained by variations in FFMI, VFL, and PMM (Table 2).

The z-structural variations (diameters, IMT) were mainly explained by variations in FFMI, regardless of the histological type of artery (Table 2). Always, (i) higher FFMI was associated with higher arterial z-structure, and, generally, (ii) FFMI was the explanatory variable with the highest relative weight [greatest explanatory ability evidenced by the B standardized (Bs) level]. Classical anthropometric indexes were not included in the explanatory z-structural models.

Variations in z-local stiffness were explained (i) by FMI (considering CFA and BA) and (ii) by PMM and VFL (considering CCA). With exception of left z-CCA EM, the BMR, BMI, BW, and BH were not included in local stiffness models. Variations in z-regional stiffness were not explained by anthropometric or body composition indexes (Table 2).

Considering aoBP and baBP, the z-BP-related parameters showed meaningful differences. Accordingly, variations in z-aoSBP were mainly explained by FFMI, but also by BMI. Variations in z-aoDBP were explained by VFL and PMM, while z-baBP was explained only by variations in BMI.

### Effect Size Analyses: Maximal Inter-Individual Arterial Variations Explained by Mono-Segmental BIA-Derived Body Composition Indexes (Aim 3)

Table 3 shows cardiovascular variations, which could be explained (regardless of other cofactors) by variations in 5, 10, 15, and 20 units of: (i) FFMI, (ii) PMM, and (iii) VFL. The expected variations are presented according to different ages (10, 30, 50, 70 years). Also, Table 3 shows the maximum cardiovascular variation (absolute and relative) that could be explained by variations in FFMI, PMM, or VFL. Figure 4 summarizes these findings.

Variations in FFMI explain variations in arterial diameters. Their absolute levels (in mm) gradually increased when considering CCA, CFA, and BA (1.5–2.0, 1.5–3.8, and 1.8–4.0 mm, respectively). Additionally, FFMI levels explain absolute variations of 0.1 to 0.3 mm in CCA IMT (Table 3). In relative terms, FFMI variations are able to explain variations in CCA, CFA, and BA diameters (30, 40, and 75%, respectively) and IMT (20–30%).

Besides, FFMI-related variations in local stiffness were different between arterial segments. FFMI-associated variations in CCA and CFA EMs reached levels of 30–60%, while, in BA, EM reached maximum levels of 130–150% (Figure 4, Table 3). This seems to indicate that there is an arterial segment-dependent “sensitivity” (CCA vs. CFA vs. BA) to changes in FFMI.

Finally, FFMI explained variations in aoSBP, but not in baBP, indicating again an “arterial segment” dependency (central vs. peripheral). Accordingly, FFMI variations explained variations of 16–22 mmHg in aoSBP, representing 17–20% relating to the MV of the reference group (Figure 4, Table 3).

PMM and VFL variations were associated with structural and stiffness variations in CCA of 25–70% (but not in the CFA or BA), and with aoDBP variations (but not in baBP) of 10–20% (Figure 4, Table 3).

### Mono- (Whole Body) and Multi-Segmental (Total, Trunk, Limbs) Fat and Fat-Free Mass Indexes: Association With Arterial Variations (Aim 4)

Supplementary File 2: Table S5 shows the subjects' characteristics assessed by multi- and mono-segmental BIA-derived approaches. It can be seen a wide age range (7–75 years), exposure to CRFs, and body composition levels (e.g., BMI: 17.1–44.6 kg/m^2^; FMI: 1.9–23.3 kg/m^2^, FFMI: 13.4–25.0 kg/m^2^). Besides, this subgroup shows wide variation in cardiovascular parameters (e.g., average z-scores between −2.1 and +3.8) (Supplementary File 2: Table S6; Supplementary File 3: Figure S1).

High agreement between the two BIA devices (Omron HF-514 vs. InBody-120) was observed in concordance and Bland-Altman analysis when comparing “fat mass” and “fat-free mass” indexes (Supplementary File 2: Table S7; Supplementary File 3: Figure S2).

Supplementary File 2: Table S8 shows correlation analyses between demographic, clinical, anthropometric, body composition, and cardiovascular z-scores characteristics.Supplementary File 3: Figures S3–S8 detail the association (“*r*” ranked from highest to lowest) between (i) cardiovascular z-scores and (i) FMI (Supplementary File 3: Figures S3–S5) or (ii) FFMI levels (Supplementary File 3: Figures S6–S8). In addition, information about the comparison of “total” marker [whole body; (IB)] and the five body segments, i.e., trunk (T), left and right arms (LA, RA), and left and right legs (LL, RL), are also provided.

For z-IMT and z-carotid diameters, the T-FMI showed the highest association, while, for z-CFA diameters, the T-FMI had the lowest level of association. The “total” (whole body) FMI achieved neither the highest nor the lowest level of association (Supplementary File 3: Figure S3). Similarly, the analysis of arterial z-stiffness showed that T-FMI followed by the “total” FMI showed the highest association regardless of the considered arterial segment (CCA, CFA, BA) (Supplementary File 3: Figure S4). Regarding the z-BP analysis (aoBP, baBP), T-FMI, and the “total” FMI had the strongest associations (Supplementary File 3: Supplementary Figure S5).

Regarding the CCA, CFA, and BA z-diameters, the “total” (whole body) and “upper limb” FFMI (RA or LA) showed the greatest levels of association (“moderate”), while the “trunk” and lower limb FFMI reached the lowest levels. Considering the z-IMT, upper limb FFMIs achieved the highest degrees of association (Supplementary File 3: Figure S6). Similarly, regarding z-local stiffness, “total” FFMI showed a greater association with CCA and CFA stiffness, while “upper limb” FFMIs showed greater associations with BA z-stiffness (Supplementary File 3: Figure S7). For the z-BP, “total” FFMI (for DBP) and “upper limb” FFMI (for SBP) were found to have the highest association, while lower limb FFMIs showed the lowest levels of association (Supplementary File 3: Figure S8).

## Discussion

To our knowledge, this is the first study to comprehensively evaluate the independent association (and effect size) of (i) mono- and (ii) multi-segmental BIA-derived body composition indexes (e.g., total fat, visceral fat, fat-free mass, and indexes) with the arterial system status. To this end, we have analyzed in a large sample of healthy children, adolescents, and adults (ii) several arterial pathways (elastic, transitional, and muscular; central and peripheral) and (iii) complementary hemodynamic, structural, and functional arterial parameters, using different non-invasive approaches (always considering the “gold standard” if available). The main findings can be summarized in six points, as follows:

- First, non-specific (classical) anthropometric indexes (i.e., BW, BMI, BMR) showed a high level of association with the structural, functional, or hemodynamic cardiovascular characteristics of the subject (z-scores). Furthermore, regardless of the classical index considered, the levels of association showed a specific hierarchy order: diameters and local arterial stiffness > BP (aortic and brachial) > regional arterial stiffness.
- Second, the joint association analysis of fat mass (i.e., BFM, PBF, FMI, VFL) and fat-free mass indexes (i.e., FFM, FFMI) with cardiovascular z-scores showed that all significant associations were positive. In other words, the higher the levels of body composition indexes, the higher levels of z-score (i.e., a greater starting point from the MV that is expected for a subject of similar age, who is not exposed to traditional CRFs).

Our study provides further evidence about the relationship between arterial characteristics (i.e., functional, structural, and hemodynamic properties) and body composition variables (i.e., FM and FFM indexes). Although there are no studies that jointly analyze BIA-derived body composition variables with arterial properties, some studies have analyzed them individually. Our results share similarities with the findings reported by Czernichow et al. (35) who reported an association between CCA IMT and body composition variables (i.e., BMI, PBF, FM, and FFM). Although, in our analysis, the structural arterial characteristics were the ones showing the highest levels of association, functional arterial parameters (mainly regional arterial stiffness) were not significantly explained by anthropometric or composition variables. These findings further support the aforementioned study, as no associations were observed by the authors between both FM and FFM and regional arterial stiffness after adjusting for covariates (35).

When analyzing the PBF and FMI individually, our results show that FMI had a stronger association with the arterial parameters than PBF. This finding was in line with Ortega et al. (10) and confirms previous studies that reported that FMI was a better predictive index than PBF for both metabolic syndrome and cardiovascular mortality (10, 36). Furthermore, FMI was shown to be strongly associated with high BP and arterial stiffness in children, adolescents (11, 37), and adults (38).

The joint analysis of FM and FFM also showed that these indexes were meaningfully associated with arterial characteristics, and, in turn, increased levels of these indexes could be indicators of elevated cardiovascular risk. Indeed, Ortega et al. showed prospectively that higher levels of FM and FFM were also predictors of greater cardiovascular risk (10). Nevertheless, these findings differ from previous results reported in the literature, showing that higher levels FFM were protective, associated with a decreased mortality risk (39, 40).

- Third, simple correlation analysis showed that fat-free mass indexes exceed the association obtained with BMI and BMR, considering structural arterial z-scores. In contrast, fat mass indexes do not exceed the association with z-scores achieved by BMI and BMR.

This work adds further data to that reported by Ortega et. al (10), demonstrating that FFM and BMI may be complementary parameters. Interestingly, the independent association between both FFM and BMI and cardiovascular z-scores showed that the strength of these associations depended on the cardiovascular parameter considered. For instance, FFM exceeds BMI in z-aoSBP but not in z-baSBP. In addition, the investigators also reached similar conclusions in the sense that BMI increases according to an excess of FM plus FFM (10). This might confirm the strong association shown between BMI and cardiovascular properties, as well as its ability to predict cardiovascular mortality. Obese populations are also characterized by an increased FFM (that might be partially explained by higher blood volume) and might lead to the need of higher stroke volumes and cardiac outputs to match metabolic demands than non-obese peers. Those characteristics might represent an extra burden for the cardiovascular system, increasing the risk of heart disease (41, 42). In fact, not only the excess of FM is considered a CRF, but also FFM (43–45). In this regard, FFM has been considered a significant determinant of BP (44, 46), regional arterial stiffness (46), CCA IMT, and lumen area (35, 47). As previously mentioned, these findings are in contradiction with previous results, which did not show significant associations between FFM and structural parameters (e.g., IMT) and cardiovascular risk (39, 40, 48).

- Fourth, multivariate analysis indicated that, regardless of age, sex, CRFs, and classic anthropometric indexes (i.e., BMI, BMR, BW, BH), variations in cardiovascular z-scores can be explained by levels of FFMI, VFL, and PMM. Independently of both CRFs and classical indexes, FFMI explains mostly the inter-individual variations in (i) CCA IMT, (ii) diameters, and local arterial stiffness regardless of the arterial type and (iii) aoSBP.

It is worth mentioning that, in multiple linear regression models, variations in structural z-scores were mainly explained by either FFMI or z-aoSBP regardless of age, sex, presence of CRFs, and classical anthropometric indexes. In fact, variations in FFMI are able to explain variations in BP levels and CCA, CFA, and BA diameters. Our data point toward an association between FFMI and impaired arterial properties, which is in line with some but not all, recent reports. For instance, FFMI was strongly associated with cardiovascular conditions, such as hypertension, peripheral and coronary artery disease in adults aged 40–69 years (49). Interestingly, in a cross-sectional study of healthy Chinese children and adolescents *n* = 1,609, median age and interquartile range: 12.86 and 5.31 years, respectively (57.6% girls)], He et al. found that the effect size of the association between body composition and baBP differed in different age ranges (12). Accordingly, FFMI was positively associated with baSBP in 9–12 years and in 15–16 years age ranges but was not significantly associated with baDBP in any age range. Verma and Sinah. (50) reported similar results as previously mentioned in a randomized cross-sectional study in children and adolescents (*n* = 733; 10–18 years). In this study, FFMI and FMI were both positively correlated with BP, being FFMI the parameter that correlated most strongly with baBP.

Our findings significantly differ from previously published data, which showed a stronger association between FMI and baBP rather than with FFMI (51). It should be noted that, in this study, the anthropometric assessment differed from what was used by other investigators. More specifically, fat percentage, FMI, and FFMI were calculated from skinfolds thickness assessment rather than from bioelectrical impedance analysis (51).

- Fifth, regardless of the body segment considered (trunk, lower and upper limbs), levels of association between FMI and cardiovascular z-scores did not exceed those found with both classic anthropometric and fat-free mass indexes. However, total body fat mass and trunk indexes [T-FMI and FMI (IB)] showed a greater strength of association with cardiovascular z-scores than the FMI of upper and lower limbs.

Our data suggest that variations in total body fat mass or central fat mass (trunk) are associated (albeit weakly) with changes in arterial properties. Evidence has suggested that trunk fat mass as well as abdominal obesity should be considered as real CRFs (35, 37, 52, 53). Indeed, trunk fat mass and abdominal fat have shown a strong correlation between each other in adult women (54). Our results are also in line with previous findings where higher arterial stiffness was associated with high-trunk FMI in children, adolescents, and adults (11, 55, 56). Furthermore, it has been found that adolescents with higher fat trunk levels demonstrated a higher risk of developing cardiovascular disease at 26 and 36 years (57, 58). Considering that arterial stiffness is an early marker of atherosclerotic disease, the distribution of body fat (mainly in trunk and abdomen) becomes relevant in the stratification of cardiovascular risk (35, 52). Indeed, central fatness has been recognized as a primary risk factor in cardiometabolic dysfunction (37) and an independent determinant of vascular health (55).

- Sixth, total (whole body) and upper limbs FFMI showed a higher level of association with z-diameters, z-IMT, z-local stiffness, and z-BP (surpassing almost all cardiovascular z-scores except for z-crPWV and PWV ratios) than lower limb FFMI indexes.

Although several studies have shown that fat distribution might be as relevant as total fat mass in stratifying the cardiovascular risk (11, 55), to our knowledge, no studies have found differences in FFMI of upper and lower limbs in relation to vascular properties. Accordingly, while increased levels of lower limb fat mass would work as a protective factor of cardiovascular disease in children, adolescents, and adults (53), increased arm fat mass was strongly associated with CRFs in women (59). Further studies, considering segmental body composition characteristics and cardiovascular properties are needed to further clarify these observations.

### Importance of Results in Clinical and Epidemiological Settings

From data obtained in healthy children, adolescents and adults, our work provides evidence on which BIA-derived indexes have the highest independent levels of association with inter-subject hemodynamic, structural, and functional arterial variabilities (deviation from expected values). Knowing to what extent BIA-derived indexes are associated with arterial properties, with independence on other subjects' characteristics and exposure to CRFs (e.g., age or sex), would be useful to define the values of arterial properties expected in association with (explained by) data obtained on body composition. This information would be of value in both the research field (e.g., when selecting variables to assess in epidemiological studies aimed at evaluating the relationship between body composition and cardiovascular health) and clinical practice (e.g., to analyze the health impact of certain conditions and/or interventions on body composition). In this regard, it would be particularly important for professionals involved in physical activity and health (e.g., in the field of nutrition, exercise/sports, medicine) to know BIA-derived body-composition indexes and/or parameters with the greatest predictive capacity for cardiovascular status, since that would be useful in terms of assessment, diagnosis, definition of interventions' objectives and strategies (as well as in their evaluation and follow-up). In this regard, the following findings and contributions should be considered.

First, regardless of other subjects' characteristics, in children, adolescents, and adults, FFMI, VFL, and PMM were the BIA-derived indexes independently associated with arterial characteristics. This adds support to the proposal that, in healthy subjects, from the general population, fat-free mass-related indexes would be equally or even more valuable in terms of a predictive capacity when compared to classical anthropometric and fat mass indexes. Then, interventions (e.g., physical training) aimed at modifying (specifically) muscle mass levels and, consequently, FFMI and/or PMM could impact positively and directly (independently) the cardiovascular system. Therefore, being aware of which and to what extent variations in body composition during actions aimed at improving physical fitness (e.g., physical activity and/or dietary programs) are associated with cardiovascular health could contribute to improved professional performance.

Second, structural characteristics of central arteries (i.e., CCA, IMT, and diameters) would be the most sensitive to variations (differences) in BIA-derived indexes (e.g., FFMI). Therefore, analysis of central (e.g., CCA) rather than peripheral (e.g., CFA and BA) arteries would be more valuable for tracking differences in arterial characteristics associated with body composition indexes. In turn, “local” arterial stiffness parameters (e.g., CCA EM) would be more sensitive than the “regional” ones in terms of association with variations in BIA-derived body composition indexes. In this regard, it is noteworthy that we found that regional arterial stiffness assessed (as in several clinical studies) through the cfPWV was not strongly associated with variations in body composition and would not be considered of choice when assessing the association of BIA-derived body composition indexes and cardiovascular status. The above add to the proposal that body composition would not homogeneously impact the arterial system but would differentially affect the arterial territories and properties. Consequently, when analyzing and discussing the impact of body composition on arterial function (e.g., arterial stiffness), it is necessary to specify the territory and parameter evaluated.

Finally, “classic” (e.g., BMI) and “new” indexes (e.g., BIA-derived FFMI) could provide complementary explanatory information. Thus, both types of indexes should not be seen in all cases as “competitors.” The above reinforces the value of classical anthropometric measurements and BIA-derived recordings to comprehensively assess the association between body composition and arterial characteristics.

### Strengths and Limitations

This work has strengths and limitations that should be considered. First, our study included a comprehensive non-invasive evaluation of arterial properties (including analysis of different histological types of arteries), obtained from a large population sample of children, adolescents, and adults. Second, the number of subjects and the statistical approach (e.g., a bootstrapping technique) were designed to increase the reliability and to analyze the association between BIA-derived body composition indexes and cardiovascular characteristics with independence of other CRFs, classical anthropometric indexes, and regardless of other body composition indexes. However, although we adjusted for several covariates, we cannot rule out the possibility of residual confounding factors that could have influenced our results. Third, having a reference group enabled us to determine through mathematical adjustments the MV, SD, and variations in cardiovascular z-scores. Since the reference group included Uruguayan children, adolescents, and adults non-exposed to CRFs, we avoided using bibliographical data from subjects who do not necessarily present characteristics similar to those of the Uruguayan population. Fourth, body composition data were corroborated using two validated BIA devices (InBody-120; OMRON-HBF514C), which showed a good concordance correlation (60–62).

We are aware that our research may have limitations: First, it is a cross-sectional study, so the causal relationship between BIA-derived body composition indexes and cardiovascular properties could not be explored. Second, information on the waist-hip ratio and neck circumferences was not included since there was no reliable information for all the subjects. Third, the body composition assessment was performed by BIA devices, a technology which is not considered the “gold standard” method for measuring body composition such as dual-energy X-ray absorptiometry or magnetic resonance imaging. Yet, these advanced imaging modalities are more expensive and operator dependent. Nowadays, BIA devices are a low cost and reliable method widely used in clinical and epidemiological settings to measure FM and FFM parameters (63) (Supplementary File 1). Finally, despite the fact that two commercial BIA devices (validated and widely used) were used in the present study, it is worth noting that the equations that allowed BIA-derived indexes to be obtained were not derived from studies in the Uruguayan population. As the other authors have done for specific populations in South America (64, 65), further studies will allow the equations for obtaining BIA-derived indexes to be evaluated and validated in the Uruguayan population.

## Conclusions

First, non-specific (classical) anthropometric indexes (BW, BMI, BMR) showed a high association with cardiovascular z-scores. Furthermore, regardless of the classical index considered, the levels of association showed a specific hierarchy order: diameters and local arterial stiffness > BP (aortic and brachial > regional arterial stiffness.

Second, the joint association analysis between both fat mass and fat-free mass indexes and cardiovascular z-scores showed that all significant associations were positive. The higher the levels of these indexes, the greater the deviation toward positive values of arterial characteristics (e.g., higher CCA IMT, DD, and/or local stiffness).

Third, fat-free mass indexes exceeded the association obtained with BMI and BMR, considering structural arterial z-scores. In contrast, fat mass indexes did not exceed the association with z-scores achieved by BMI and BMR.

Fourth, regardless of age, sex, classical CRFs and anthropometric indexes, variations in arterial z-scores can be mainly explained by levels of (i) FFMI, (ii) VFL, and (iii) PMM. FFMI explains mostly inter-individual variations in (i) CCA IMT, (ii) diameters and local arterial stiffness regardless of the arterial type, and (iii) aoSBP.

Fifth, regardless of the body segment considered (trunk, lower and upper limbs), levels of association between FMI and arterial z-scores did not exceed those found with both classic anthropometric and fat-free mass indexes. However, total body fat mass and trunk indexes showed a greater strength of association with cardiovascular z-scores than the FMI of upper and lower limbs.

Sixth, total and upper limb FFMI showed a higher level of association with z-diameters, z-IMT, z-local stiffness, and z-BP than lower limb FFMI indexes.

## Data Availability Statement

The raw data supporting the conclusions of this article will be made available by the authors, without undue reservation.

## Ethics Statement

The studies involving human participants were reviewed and approved by Comités de Ética del Hospital de Clínicas, Instituto Superior de Educación Física, and Centro Hospitalario Pereira-Rossell (Universidad de la República; Uruguay). Written informed consent to participate in this study was provided by the participants' legal guardian/next of kin.

## Author Contributions

MG-G, JT, DB, and YZ contributed to conception and design of the study, performed the anthropometric, body composition, and cardiovascular non-invasive recordings, constructed and organized the database, and performed the statistical analysis. MG-G, JT, MP, DB, and YZ wrote the first draft and final version of the manuscript, contributed to the manuscript revision, read, and approved the submitted version. All authors contributed to the article and approved the submitted version.

## Funding

This research was funded by Programa Desarrollo de las Ciencias Básicas (PEDECIBA, Ministerio de Educación y Cultura, Universidad de la República), Agencia Nacional de Investigación e Innovación (ANII), grant number PRSCT–008–020; and extra budgetary funds provided by DB, YZ, and CUiiDARTE Centre.

## Conflict of Interest

The authors declare that the research was conducted in the absence of any commercial or financial relationships that could be construed as a potential conflict of interest.

## Publisher's Note

All claims expressed in this article are solely those of the authors and do not necessarily represent those of their affiliated organizations, or those of the publisher, the editors and the reviewers. Any product that may be evaluated in this article, or claim that may be made by its manufacturer, is not guaranteed or endorsed by the publisher.

## Abbreviations

aoBP: Aortic blood pressure
aoDBP: Aortic diastolic blood pressure
aoSBP: Aortic systolic blood pressure
BA: Brachial artery
baDBP: Brachial artery diastolic blood pressure
baMBP: Brachial artery mean blood pressure
baSBP: Brachial artery systolic blood pressure
BH: Body height
BIA: Bioelectrical impedance analysis
BMI: Body mass index
BMR: Basal metabolic rate
BP: Blood pressure
BW: Body weight
CCA: Common carotid artery
CFA: Common femoral artery
cfPWV: Carotid-femoral pulse wave velocity
CRFs: Cardiovascular risk factors
crPWV: Carotid-radial pulse wave velocity
DD: End-diastolic arterial diameter
EM: Elastic modulus
FFMI: Fat-free mass index
FMI: Fat mass index
IB: In body [multi-frequency (20 kHz and 100 kHz) and multi-segmental bioelectrical impedance device (InBody-120, InBody Co., Seoul Korea)].
IMT: Intima-media thickness
MV: Mean value
OM,: Omron [mono-frequency (50 kHz) and mono-segmental bioelectrical impedance device (HBF-514C (OM), Omron Healthcare, Inc., Illinois USA)]
PBF: Body fat percentage
PWV: Pulse wave velocity
PWV Ratio: Pulse wave velocity ratio (cfPWV/crPWV quotient)
SCOR: SphygmoCor-CvMS device
SD: Standard deviation
SysD: Peak systolic arterial diameter
VFL: Visceral fat level
z-: z-score
β: Beta (stiffness) index.
