# Supplementary material for: Fat-Free Mass Index, Visceral Fat Level, and Muscle Mass Percentage Better Explain Deviations From the Expected Value of Aortic Pressure and Structural and Functional Arterial Properties Than Body Fat Indexes
Source: Front Nutr. 2022 Apr 29;9:856198. doi: 10.3389/fnut.2022.856198 (PMC9099434; doi:10.3389/fnut.2022.856198)
Supplement: Supplementary file 1 [file Data_Sheet_1.docx]

# Supplementary File 1

# Devices: technical characteristics

## Omron HBF-514C, Omron Healthcare, Inc., Illinois, USA

This device, frequently called ´Full Body Sensor Body Composition Monitor and Scale´ passes a weak electrical current of 50 kHz (mono-frequency) and less than 500 µA through the body when taking a measurement to determine mono-segmental body composition. Unlike other devices that rely on “foot-to-foot measurements”, Omron’s monitor measures whole body (“arm to foot”) which provides a clinically-proven accurate profile. It is a standing 8-point tactile electrode bioimpedance analysis (BIA) analyzer. It is designed for portability, making it a possible option when providing services to underserved and rural populations or to individuals who cannot travel to a testing site. Figure 1 exemplifies details related to its use and technical details.


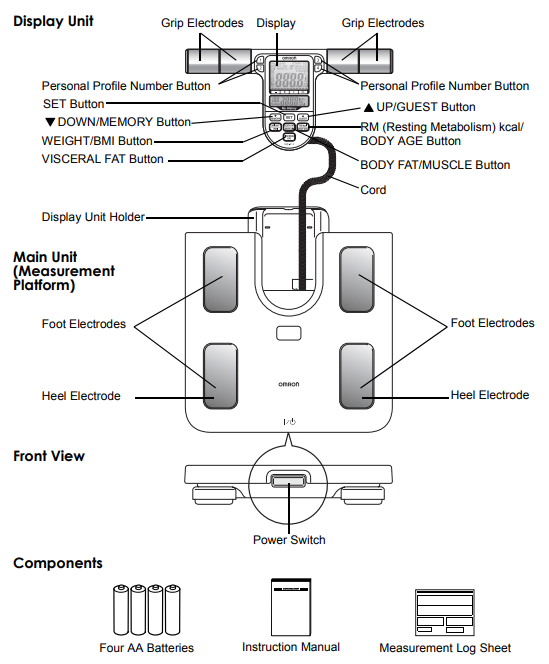

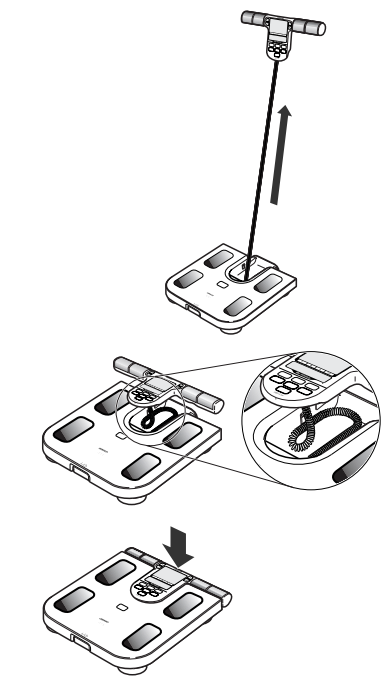


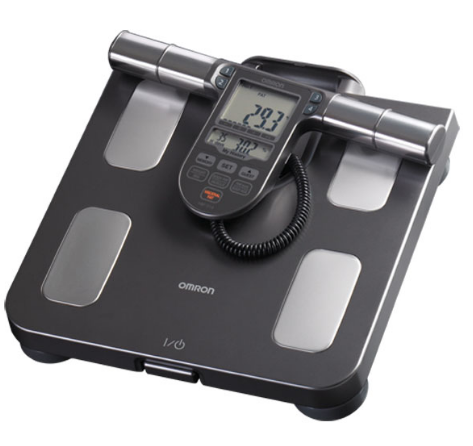

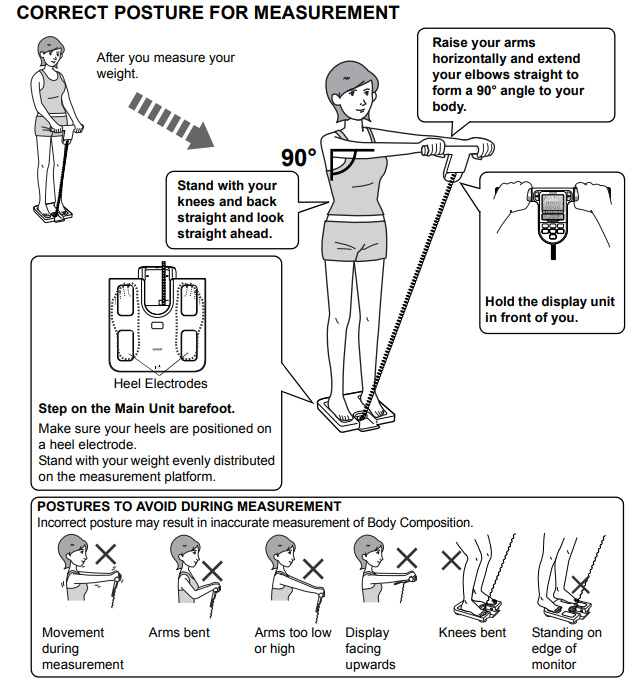


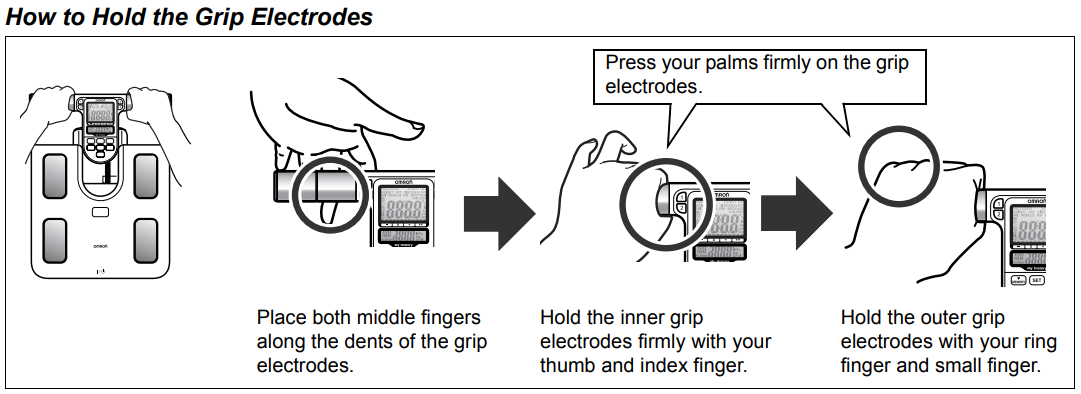


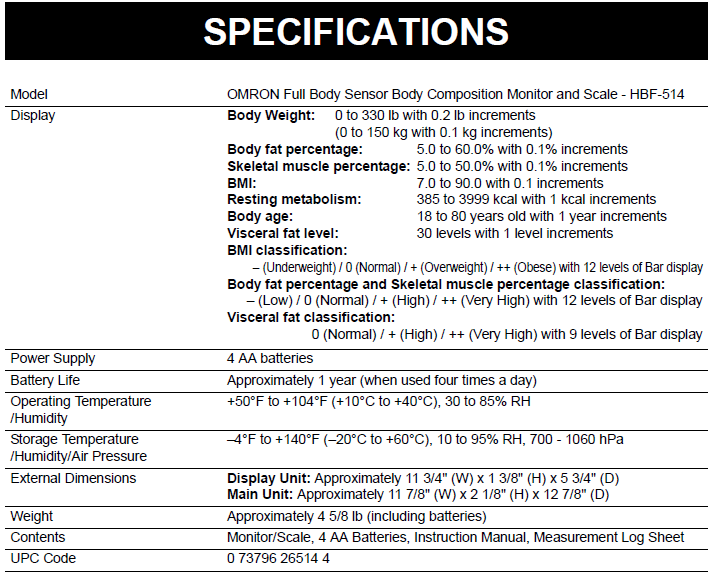


Figure 1. OMRON® HBF-514 Full Body Sensor Body Composition Monitor and Scale. Information and images provided by the supplier. Available at: <https://omronhealthcare.com/wp-content/uploads/hbf-514c-instruction-manual.pdf>

## InBody-120, InBody Co., Seoul, Korea

This device passes weak electrical currents of 20 and 100 kHz (multi-frequency) and less than 500 µA through the body when taking a measurement to determine the multi-segmental body composition. Unlike conventional BIA equipments that take partial measurements and rely upon formulas to estimate whole body composition, the InBody technique models human body as composed by 5 interconnecting cylinders and takes direct impedance measurements from body compartments. A tetrapolar 8-point tactile electrode system is used, which separately measures the impedance of the subject’s trunk, arms, and legs at two different frequencies (20 and 100 KHz). The InBody 120 body-composition analyzer has in-built hands and feet electrodes. During measurements, subjects are asked to wear normal indoor clothes and to stand barefooted in upright position with feet on the feet electrodes on the machine platform and the arms abducted with hands gripping on to the hands electrodes on the handles. Participant’s arms and legs should be slightly abducted to avoid the contact between the arms and torso and between the upper thighs. Data obtained is sent (Bluetooth) to a computer and processed on Look-In´Body 120 Software. As the Omron, the InBody device is designed for portability. Figure 2 exemplifies and gives details about its use and technical issues.


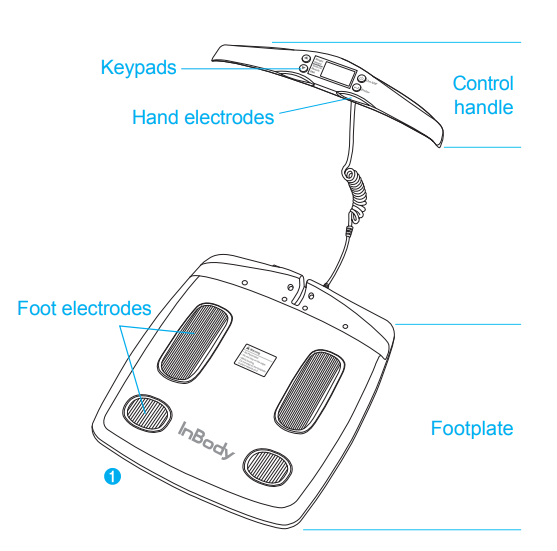

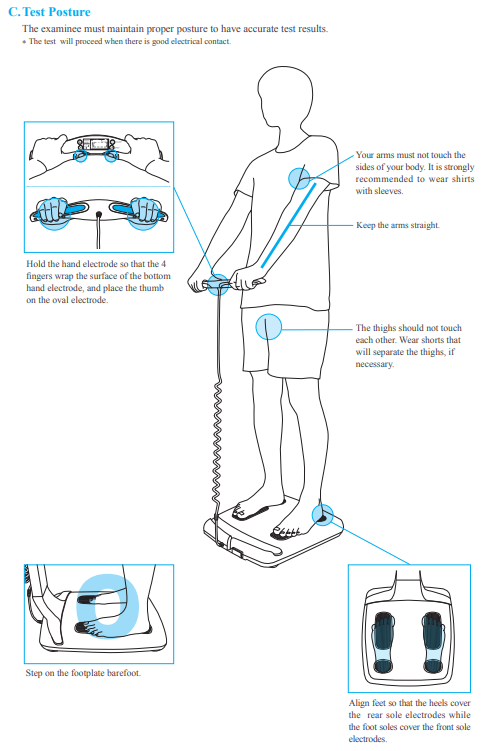


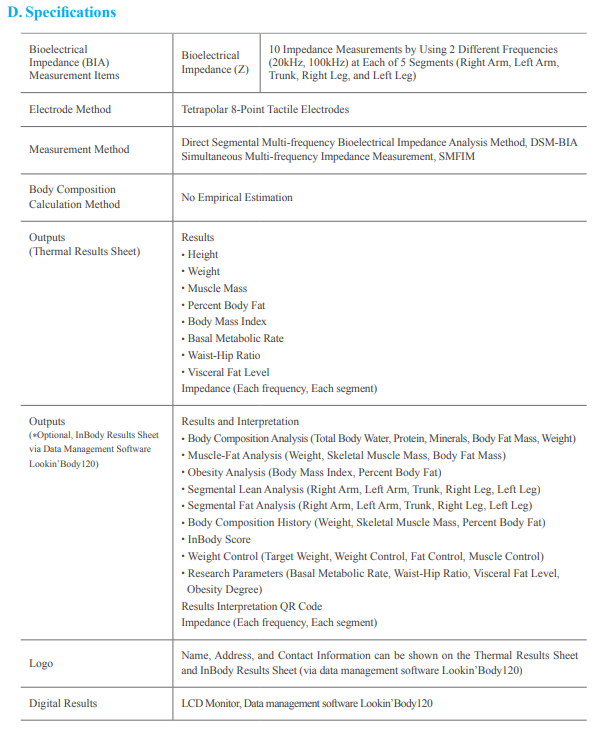

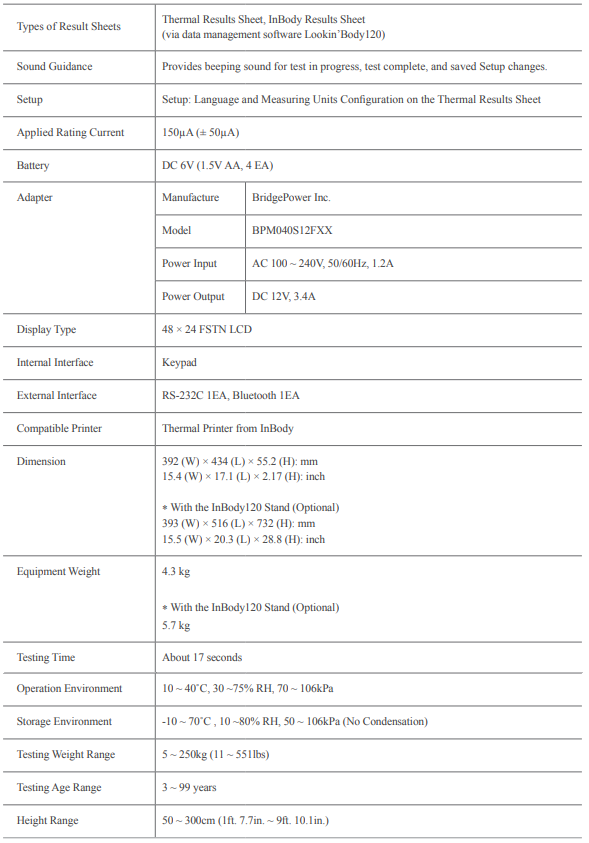


Figure 2. InBody 120. Information and images provided by the supplier. Available at: <https://nl.inbody.com/wp-content/uploads/2020/02/120_manual_ENG_E_180701.pdf>

# BIA variables measured and reported

Body weight, fat mass (FM), fat free mass (FFM), muscle mass, basal metabolic rate and visceral fat level (VFL) were measured and calculated with both devices. Fat mass (FMI) and fat-free mass (FFMI) indexes were calculated by dividing, respectively, FM and FFM by squared height. Specific data were also obtained for different segments (i.e., trunk, upper and lower limbs´ FMI and FFMI). Body mass index (BMI) was obtained as body weight divided by squared height. The percentual body fat (%BF or PBF) was obtained dividing FM by weight and multiplied by 100 (Figure 3).


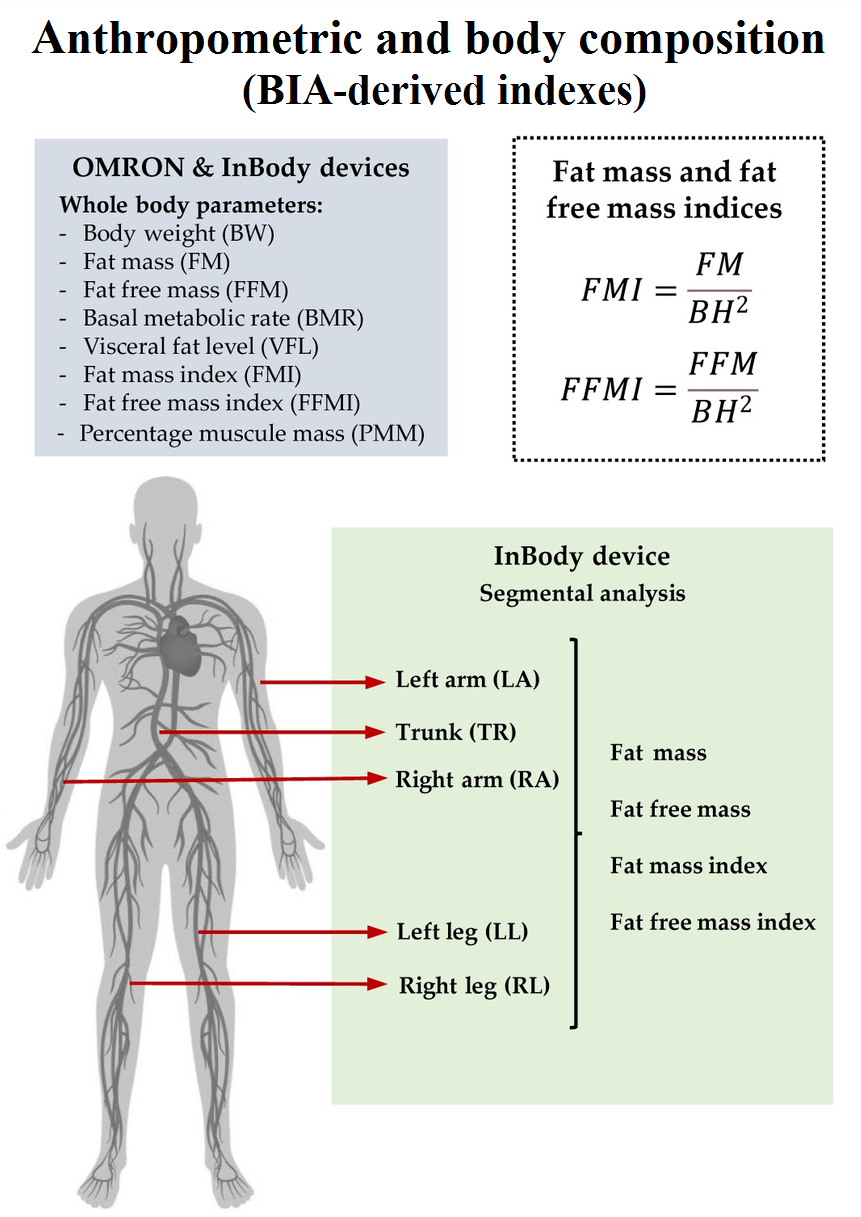


Figure 3. Anthropometric evaluation using OMRON HBF-514C device and InBody-120 device. Some variables can be measured or calculated with both devices (grey box), and others only with InBody (green box). Raw data output is calculated by the manufacturer's algorithms, included BW, FM, FFM, BMR, and VFL.

# Validity of BIA measurements

In several works, body composition measures obtained with Inbody technology were compared with those obtained with dual energy X-ray absorptiometry (DXA) and/or magnetic resonance imaging (MRI). About this, currently, DXA appears to be an accurate method for assessing body composition and has gained widespread acceptance in clinical research. This noninvasive technique can accurately determine total and segmental (i.e., trunk, leg and arm FM and FFM) body composition. However, the method is expensive and not practical in routine clinical setting or large epidemiological studies. Now DXA is increasingly being used as a validation tool for more novel techniques.

Some studies have compared body composition measurements obtained with DXA and/or MRI, and those obtained with InBody and Omron BIA devices. Their results are analyzed below.

## InBody technology vs. DXA/MRI

Gibson et al. (2008) compared %BF data from InBody BIA device and DXA (reference method) in a sample of 150 hispanic, black and white adults, of different BMI and age. The authors concluded that the strength of the correlations and the lack of significant mean differences in men would suggest the promise of BIA technology for assessing %BF in apparently healthy men. In women, the correlations were slightly stronger between InBody and DXA methods, but there were also significant differences between means and a greater trend of InBody to overestimate %BF. The authors concluded that the high reliability of the tests and the ease with which analyses can be carried out with minimal involvement suggest that InBody devices may be suitable for monitoring body composition over time.

Ling et al. (2011) examined the accuracy of Direct Segmental Multi-Frequency BIA in assessing segmental body composition in addition to whole body composition in a large representative sample of middle-aged Dutch population, using DXA as a reference standard. The subjects were participants in the Leiden Longevity Study, where 420 families consisting of long-lived Caucasian siblings together with their offspring and the partners of the offspring were recruited. Four hundred and eighty four of the offspring and their partners (female n=242; male n=242) were included. Their body composition was measured using InBody BIA and DXA. Authors reported excellent degree of agreement between the methods for the estimation of Lean Body Mass (LBM), FM and %BF in both genders (Intra-class Correlation Coefficients (ICC) ≥0.88, all p <0.001). There were also significant, albeit slightly lower degree of agreement in segmental lean mass data (ICC ≥0.83, all p <0.001). In both males and females trunk lean mass data obtained with the different approaches showed high degree of agreement (ICC > 0.70, p <0.001). Although agreement was considered excellent, there was a narrow limit of agreement in Bland-Altman for the LBM measurement and relatively wider limits for the FM and %BF measurements. Proportional bias was noted for FM measurement, with underestimation by BIA at lower FM and overestimation at higher FM (r=-0.654, p <0.001 and r=-0.571, p <0.001 for female and male, respectively). Similar bias was also noted for %BF measurement (r=-0.566, p <0.001 and r=-0.557, p <0.001 for female and male, respectively). However, overall BIA appeared to overestimate the FM and %BF on the Bland-Altman plots. On average, BIA underestimated LBM by 1.8% and overestimated FM and %BF by 8.0% and 7.0% respectively. Authors concluded that InBody would be a valid tool for the assessment of whole body composition and segmental lean mass measurements in middle-aged population when validated against DXA.

Kim et al. (2011) studied 174 healthy adults (80 men and 94 women; age: 18 to 88 years) and reported that the 8-electrode InBody device showed good agreement for the prediction of FFM and %BF in Korean adults. However, the InBody device had small, but systemic, errors in %BF and FFM in terms of the predictive accuracy for individual estimation. The total errors led to an overestimation of %BF in lean individuals among men and an underestimation of %BF among obese women. Authors, recommend equations for the correction of these total errors when using InBody device in a Korean adult population.

Anderson et al. (2012) hypothesized that there would be no significant differences between DXA and InBody-derived total LBM, appendicular lean mass (ALM), trunk lean mass (TM), and total FM. They included 25 men and 25 women (age: 18 to 49 years, body weight: 73.6±15.4 kg). Healthy, lean and obese individuals (according to BMI) were included. Authors reported that InBody technology would be a valid estimator of LBM and FM in men and of LBM, ALM, and FM in women. In addition, InBody technology would be a valid tool to estimate TM in men and women. It was concluded that InBody technology would provide reliable estimates of body composition with a single measurement and could be used as an alternative to DXA for the analysis of specific body composition variables. In quantitative or numerical terms, authors reported that InBody overestimated LBM in women (approximately (aprox.) 2.5 kg, p <0.001) and underestimated ALM in men (aprox. 3.0 kg, p <0.05) and women (aprox. 1.0 kg, p <0.05). The InBody device overestimated FM in men (1.6 kg, p <0.05) and underestimated TM in women (0.6 kg, p ≤0.05). Regression analyses in men showed R^2^ (0.87-0.91), standard error (SE) 2.3-2.8 kg, and limits of agreement (LOAs; 4.5-5.7 kg) for LBM; R^2^ (0.62-0.87), SE (1.5-2.6 kg) and LOA (3.2-6.0 kg) for ALM; R^2^ (0.52-0.71), SE (2.4-3.0 kg) and LOA (4.6-6.1 kg) for TM; and R^2^ (0.87-0.93), SE (1.9-2.6 kg), and LOA (5.9-6.2 kg) for FM. Regression analyses in women revealed R^2^ (0.87-0.88), SE (1.8-1.9 kg), and LOA (4.1-4.2 kg) for LBM; R^2^ (0.78-0.79), SE (1.4-1.5 kg), and LOA (2.7-2.9 kg) for ALM; R^2^ (0.76-0.77), SE (1.0 kg), and LOA (2.2-2.3 kg) for TM; and R^2^ (0.95), SE (2.2 kg), and LOA (4.3-4.4 kg) for FM. Additionally, the authors reported that InBody technology displayed high test-retest reliability. ICC was greater than 0.99 in all cases and the percent coefficient of variation was, on average, 1.5 % for men and women, indicating strong repeatability.

Karelis et al. (2013) studied 145 healthy subjects (age: 44.6±20 years; men=51; women =94; BMI= 24.5±3.8 kg/m^2^) volunteers recruited from the University of Quebec at Montreal and Montreal communities. The authors investigated the convergent validity of InBody device with DXA. The results showed strong significant correlations between methods for FM, %BF, total FFM and trunk FFM (r=0.94 to 0.99). Furthermore, authors showed modest but significant correlation between methods for appendicular FFM (r=0.63). No significant biases (Bland-Altman test) were observed between Inbody and DXA for FM, %BF, and total FFM. In turn, trunk and appendicular FFM were shown to have significant biases between Inbody and DXA. Authors concluded that portable Inbody technology may be an acceptable device to measure FM, %BF and total FFM (except for women) in healthy adults. There appears to be a systematic bias for the estimation of trunk and appendicular FFM when using the Inbody device.

Recently McLester et al. (2020), evaluated the reliability of several InBody devices to measure %BF, FM, and FFM in the general population, and compared results with DXA. A total of 31 males and 36 females participated in two days of testing (24-72 hours apart). The authors reported that InBody devices were reliable in men and women as indicated by high ICC for %BF (0.98), FM (0.98) and FFM (0.99) and low SE of measurement for %BF (0.77%, 0.99%), FM (0.54, 0.87 kg), and FFM (0.58, 0.84 kg) and minimum difference for %BF (2.12 %, 2.73 %), FM (1.49, 2.39 kg), and FFM (1.60, 2.32 kg), respectively. Data from InBody and DXA showed systematic bias (%BF and FM underestimation; FFM overestimation) for all comparisons (p <0.05). Proportional bias was observed for FM in women and FFM in men. There was small individual error for all comparisons as indicated by the SE of estimate and 95% LOAs. The authors concluded that InBody analyzers would show small individual errors and could be used as a surrogate when DXA is not available.

## Omron technology vs. DXA/MRI

Wang et al. (2013) compared Omron technology (HBF-359) with DXA and MRI methods analyzing VFL, %BF and skeletal muscle mass percentage (SMM%, or FFM for Omron device) data. Body composition was measured in 200 healthy volunteers (100 men and 100 women, mean age 48 years). Correlation coefficients between BIA and DXA or MRI ranged from 0.71 to 0.89 for %BF, SMM%, and VFL in all subjects, and in men and women separately (p <0.001 for all). Compared with DXA, Omron device underestimated %BF by 25.8% in men and 29.6% in women (p <0.001). Omron device data showed underestimations (negative) and overestimations (positive) when compared MRI. In this regard, the differences were +1.9% (p=0.02) and +1.7% (p=0.10) for SMM% and +13.3% (p<0.001) and 28.5% (p=0.006) for VFL, in men and women, respectively. Authors concluded that Omron devices would be accurate in the estimation of body composition, particularly for SMM% or FFM estimation. The authors stated that their findings support that compared with standard MRI or DXA method, Omron device would provide accurate and reliable estimations of SMM%, and less accurate estimations of %BF and VFL. In addition, Omron-estimation of VFL tended to be more closely correlated with MRI than anthropometric measurements, such as waist circumference with a correlation coefficient of 0.78 in men and women combined (vs. r=0.84, p=0.07).

Pietiläinen et al. (2013) analyzed Omron BIA device agreement with DXA and MRI in estimating FM, SMM and VFL during a 12-month body weight loss intervention. A total of 19 obese adults (12 females, 7 males) aged 20.2 to 48.6 years, mean BMI 34.6 (SE=0.6) kg/m^2^, were studied. Body fat, skeletal muscle and VFL were measured by BIA (Omron BF-500) and compared with DXA (FM and skeletal muscle) at baseline, 5 and 12 months, and with MRI (visceral fat) at baseline and 5 months. The authors reported that compared to DXA, the BIA accurately assessed fat loss (Mean value ± SE) (7.0±1.5 vs. 7.0±1.4 Kg, p=0.94) and muscle (1.0±0.2 vs. 1.4±0.3 Kg., p=0.18). While body fat data was similar, skeletal muscle was underestimated by 1 to 2 Kg using BIA at each time point. Compared to MRI, BIA overestimated visceral fat, particularly in males. BIA and DXA showed high correlations for fat estimation in both cross-sectional and longitudinal analysis (r=0.91 to 0.99). BIA would allow detecting muscle and visceral fat more accurately in cross-sectional studies (r=0.77 to 0.87 and r=0.40 to 0.78, respectively) than in longitudinal evaluations (r= 0.24 to 0.61 and r=0.46, respectively). Skeletal muscle mass may be slightly underestimated by BIA (compared with DXA). In turn, changes in skeletal muscle would be better assessed in long- than in short-term periods. Omron-derived visceral fat index captures the direction of change, but with systematic errors before and after weight loss; prediction of true visceral fat may be limited, especially in males.

# References

Anderson LJ, Erceg DN, Schroeder ET. Utility of multifrequency bioelectrical impedance compared with dual-energy x-ray absorptiometry for assessment of total and regional body composition varies between men and women. *Nutr Res.* (2012) 32(7):479-85. doi: 10.1016/j.nutres.2012.05.009.

Gibson AL, Holmes JC, Desautels RL, Edmonds LB, Nuudi L. Ability of new octapolar bioimpedance spectroscopy analyzers to predict 4-component-model percentage body fat in Hispanic, black, and white adults. *Am J Clin Nutr.* (2008) 87(2):332-8. doi: 10.1093/ajcn/87.2.332.

Karelis AD, Chamberland G, Aubertin-Leheudre M, Duval C; Ecological mobility in Aging and Parkinson (EMAP) group. Validation of a portable bioelectrical impedance analyzer for the assessment of body composition. *Appl Physiol Nutr Metab.* (2013) 38(1):27-32. doi: 10.1139/apnm-2012-0129.

Kim H, Kim CH, Kim DW, Park M, Park HS, Min SS, et al. External cross-validation of bioelectrical impedance analysis for the assessment of body composition in Korean adults. *Nutr Res Pract.* (2011) 5(3):246-52. doi: 10.4162/nrp.2011.5.3.246.

Ling CH, de Craen AJ, Slagboom PE, Gunn DA, Stokkel MP, Westendorp RG, et al. Accuracy of direct segmental multi-frequency bioimpedance analysis in the assessment of total body and segmental body composition in middle-aged adult population. *Clin Nutr.* (2011) 30(5):610-5. doi: 10.1016/j.clnu.2011.04.001.

McLester CN, Nickerson BS, Kliszczewicz BM, McLester JR. Reliability and Agreement of Various InBody Body Composition Analyzers as Compared to Dual-Energy X-Ray Absorptiometry in Healthy Men and Women. *J Clin Densitom.* (2020) 23(3):443-450. doi: 10.1016/j.jocd.2018.10.008.

Pietiläinen KH, Kaye S, Karmi A, Suojanen L, Rissanen A, Virtanen KA. Agreement of bioelectrical impedance with dual-energy X-ray absorptiometry and MRI to estimate changes in body fat, skeletal muscle and visceral fat during a 12-month weight loss intervention. *Br J Nutr*. (2013) 109(10):1910-6. doi: 10.1017/S0007114512003698.

Wang JG, Zhang Y, Chen HE, Li Y, Cheng XG, Xu L, et al. Comparison of two bioelectrical impedance analysis devices with dual energy X-ray absorptiometry and magnetic resonance imaging in the estimation of body composition. *J Strength Cond Res.* (2013) 27(1):236-43. doi: 10.1519/JSC.0b013e31824f2040.

# Acronyms

| Acronym | Definition |
| --- | --- |
| %BF | Percentage body fat |
| ALM | Appendicular lean mass |
| BIA | Bioimpedance analysis |
| BMI | Body mass index |
| DXA | Dual energy X-ray absorptiometry |
| FFM | Fat-free mass |
| FFMI | Fat-free mass index |
| FM | Fat mass |
| FMI | Fat mass index |
| ICC | Intra-class correlation coefficients |
| LBM | Lean body mass |
| LOAs | Limits of agreement |
| MRI | Magnetic resonance imaging |
| SE | Standard error |
| SMM% | Skeletal muscle mass percentage |
| TM | Trunk lean mass |
| VFL | Visceral fat level |
