# Supplementary material for: Fat-Free Mass Index, Visceral Fat Level, and Muscle Mass Percentage Better Explain Deviations From the Expected Value of Aortic Pressure and Structural and Functional Arterial Properties Than Body Fat Indexes
Source: Front Nutr. 2022 Apr 29;9:856198. doi: 10.3389/fnut.2022.856198 (PMC9099434; doi:10.3389/fnut.2022.856198)
Supplement: Supplementary file 3 [file Data_Sheet_3.docx]

# Supplementary File 3

**
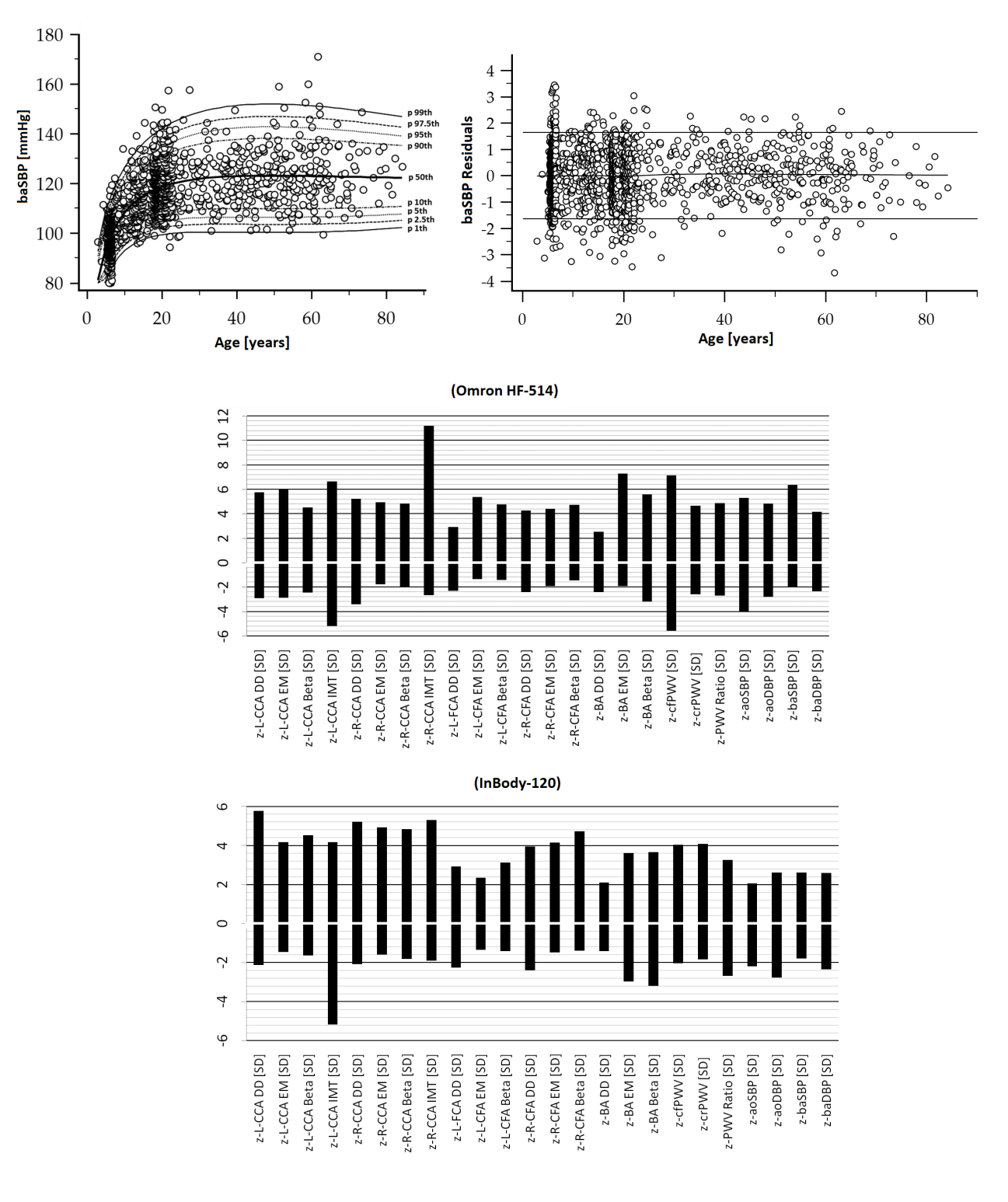
**

**Figure S1**. Top: Age-related profiles (1st, 2.5th, 5th, 10th, 50th, 90th, 95th, 97.5th and 99th percentiles) for baSBP (left) and residuals for the analyzed variable (right). Middle and Bottom: Range of variation of cardiovascular z-scores from group of children, adolescents and adults evaluated with mono-segmental (Omron) and multi-segmental (InBody) BIA devices. Abbreviations as in text.


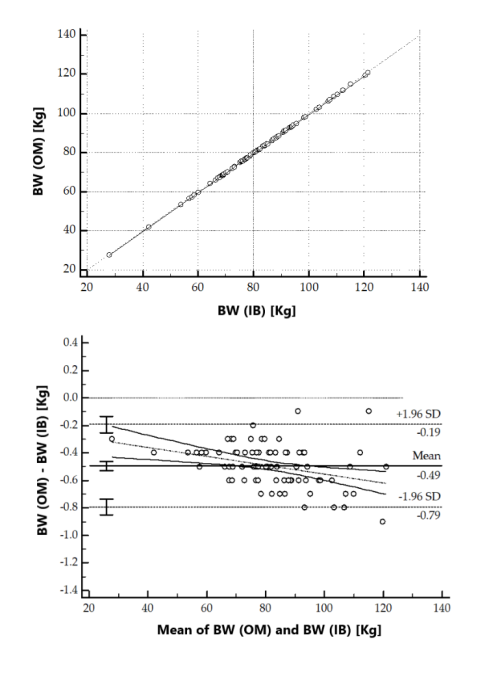
**
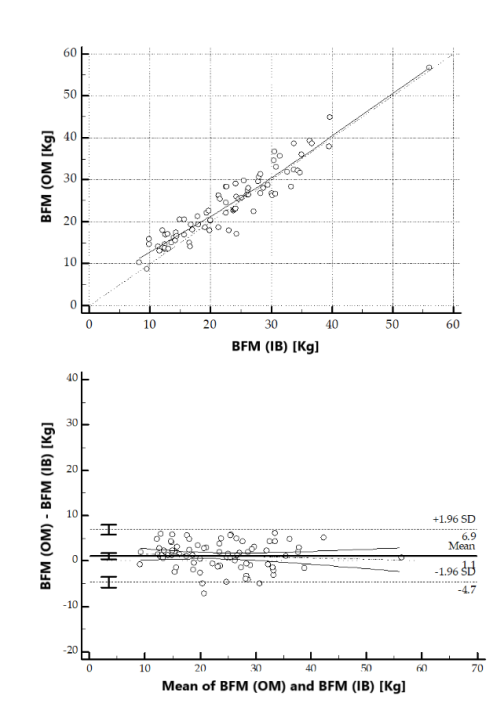

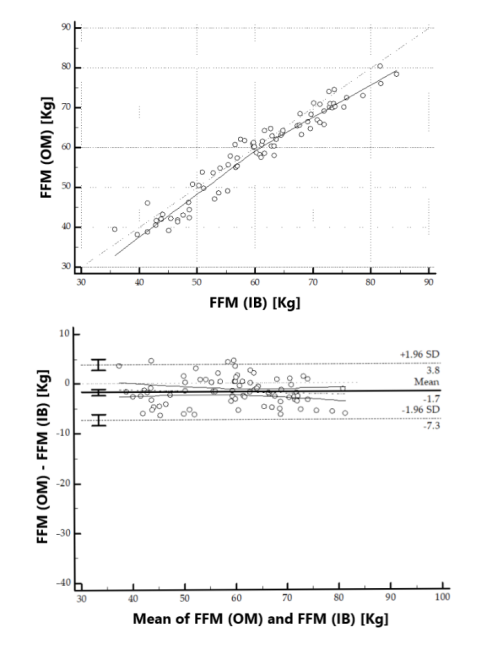

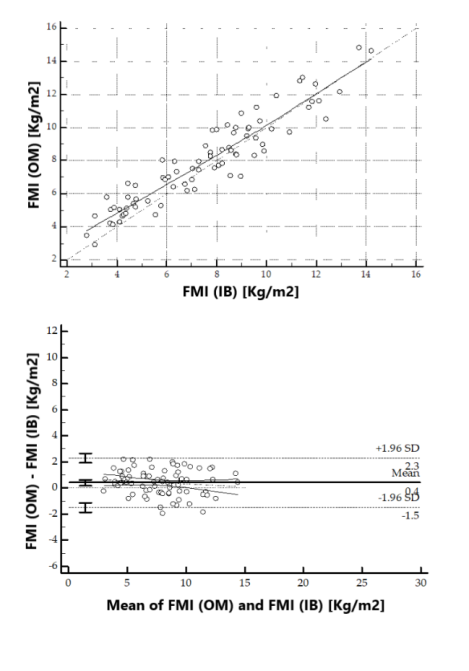

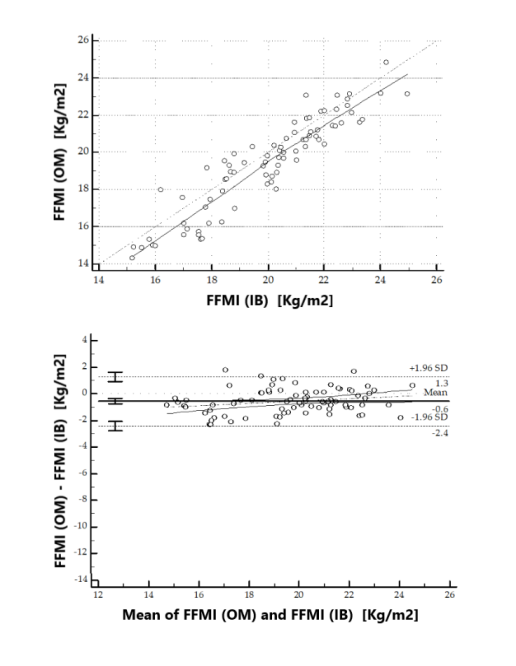
**

**Figure S2.** Concordance correlation coeficient and Bland-Altman analysis of BW, BFM, FFM, FMI and FFMI (OMRON HBF-514C vs. InBody devices). BW: body weight; BFM: body fat mass; FFM: fat free mass; FMI: fat mass index; FFMI; fat free mas index; OM: OMRON device; IB: InBody device; SD: standard deviation.


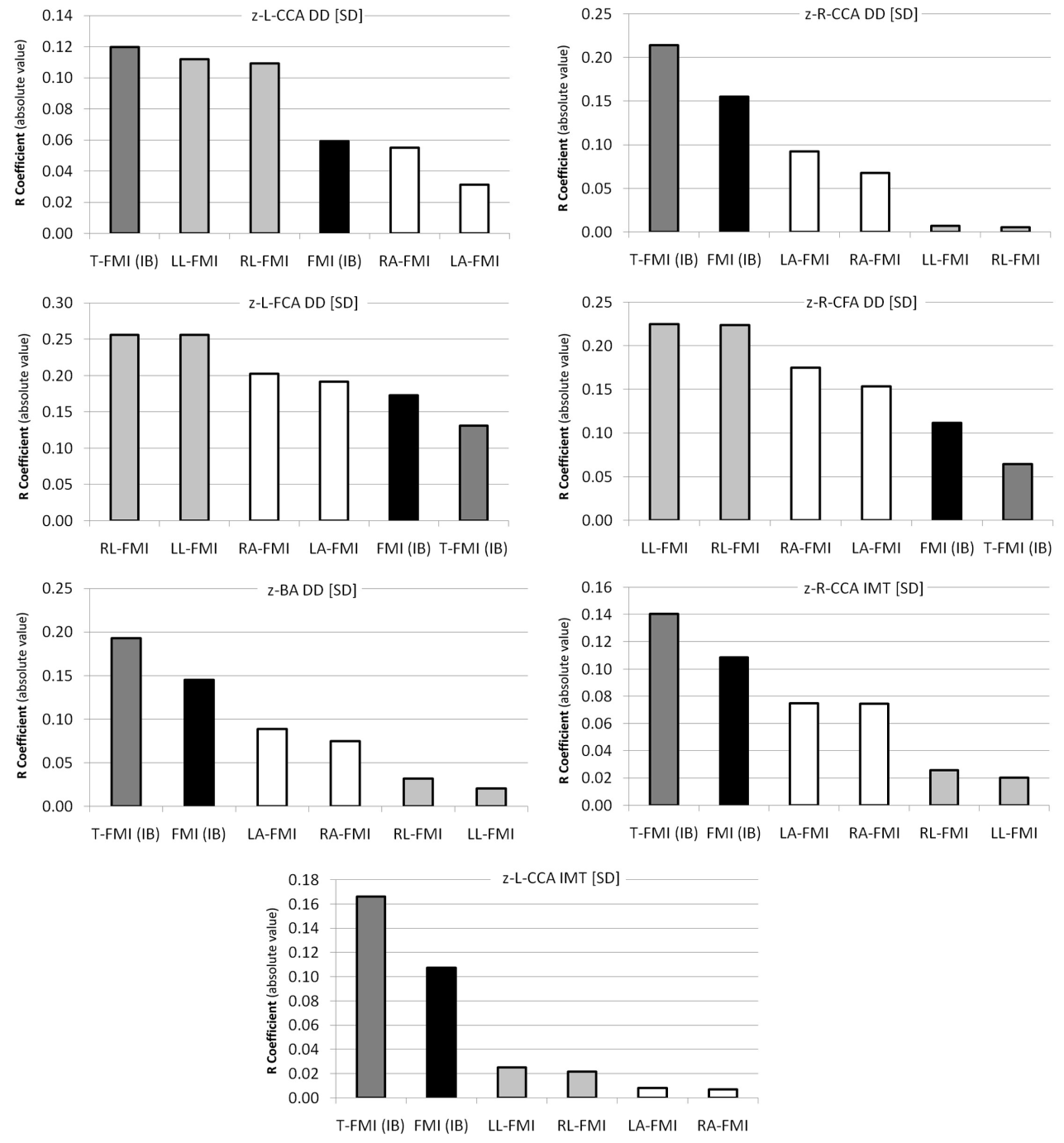


**Figure S3**. Comparison of the levels of association (´r´, absolute value ranked from highest to lowest) between structural z-scores (diameters and IMT) and FMI levels obtained for: (i) whole body (FMI[IB]), (ii) trunk (T-FMI), (iii) right and left upper limbs (RA-FMI, LA-FMI), and (iv) right and left lower limbs (RL-FMI, LL-FMI). Abbreviations as in text.


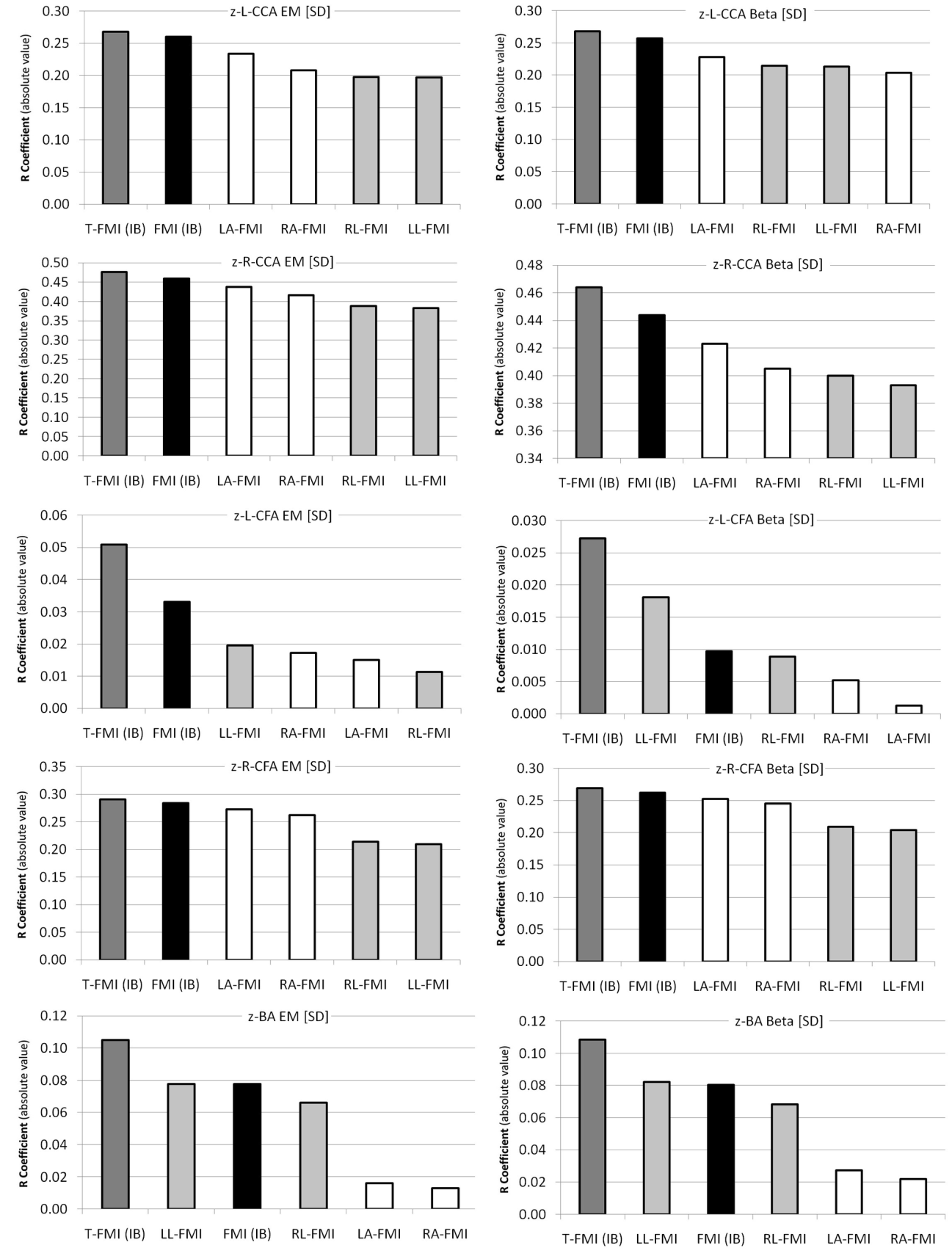


**Figure S4**. Comparison of the levels of association (´r´, absolute value ranked from highest to lowest) between local stiffness z-scores (i.e., EM and Beta Index) and FMI levels obtained for: (i) whole body (FMI[IB]), (ii) trunk (T-FMI), (iii) right and left upper limbs (RA-FMI, LA-FMI), and (iv) right and left lower limbs (RL-FMI, LL-FMI). Abbreviations as in text.


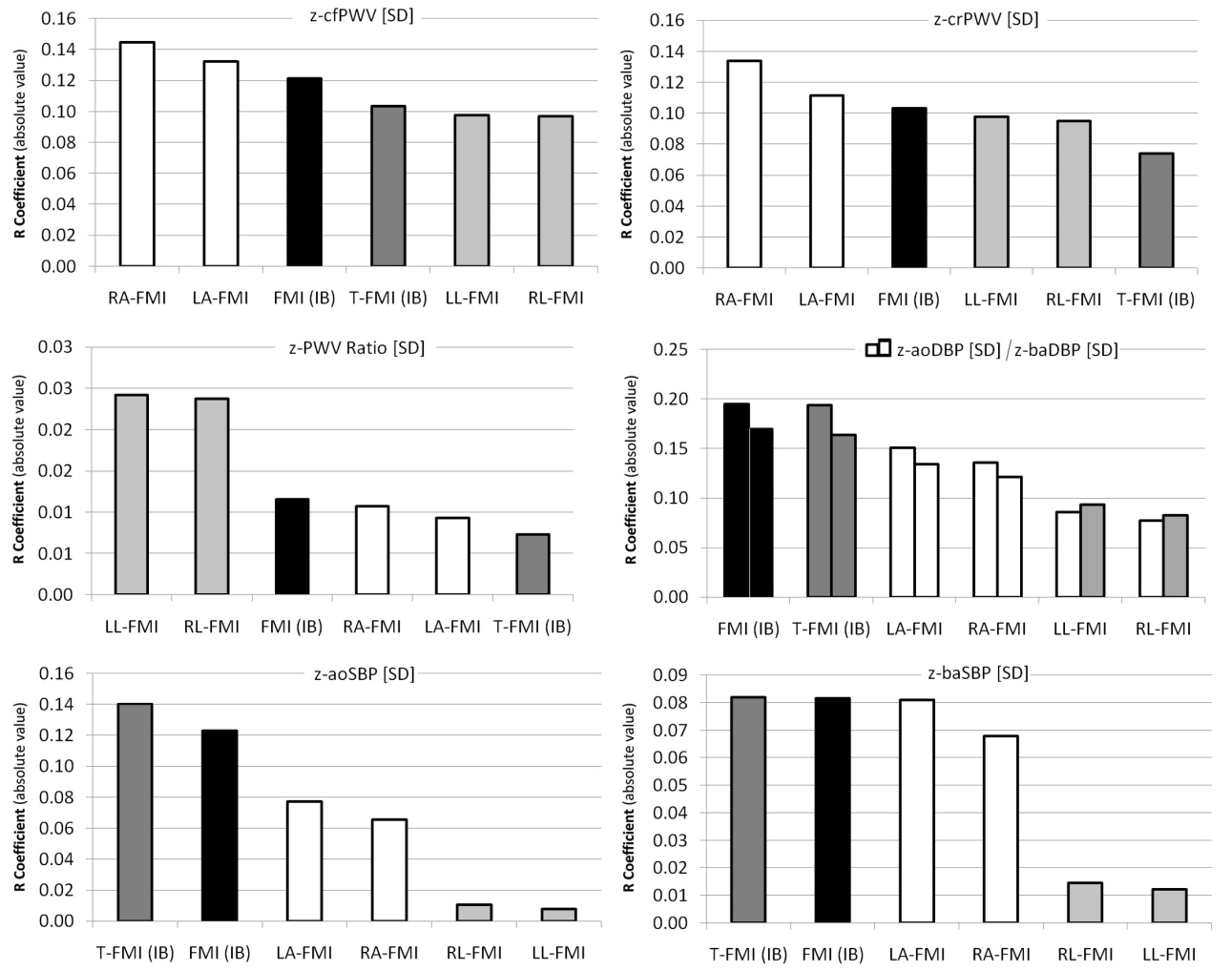


**Figure S5**. Comparison of the levels of association (´r´, absolute value ranked from highest to lowest) between regional stiffness z-scores, BP z-scores and FMI levels obtained for: (i) whole body (FMI[IB]), (ii) trunk (T-FMI), (iii) right and left upper limbs (RA-FMI, LA-FMI), and (iv) right and left lower limbs (RL-FMI, LL-FMI). Abbreviations as in text.


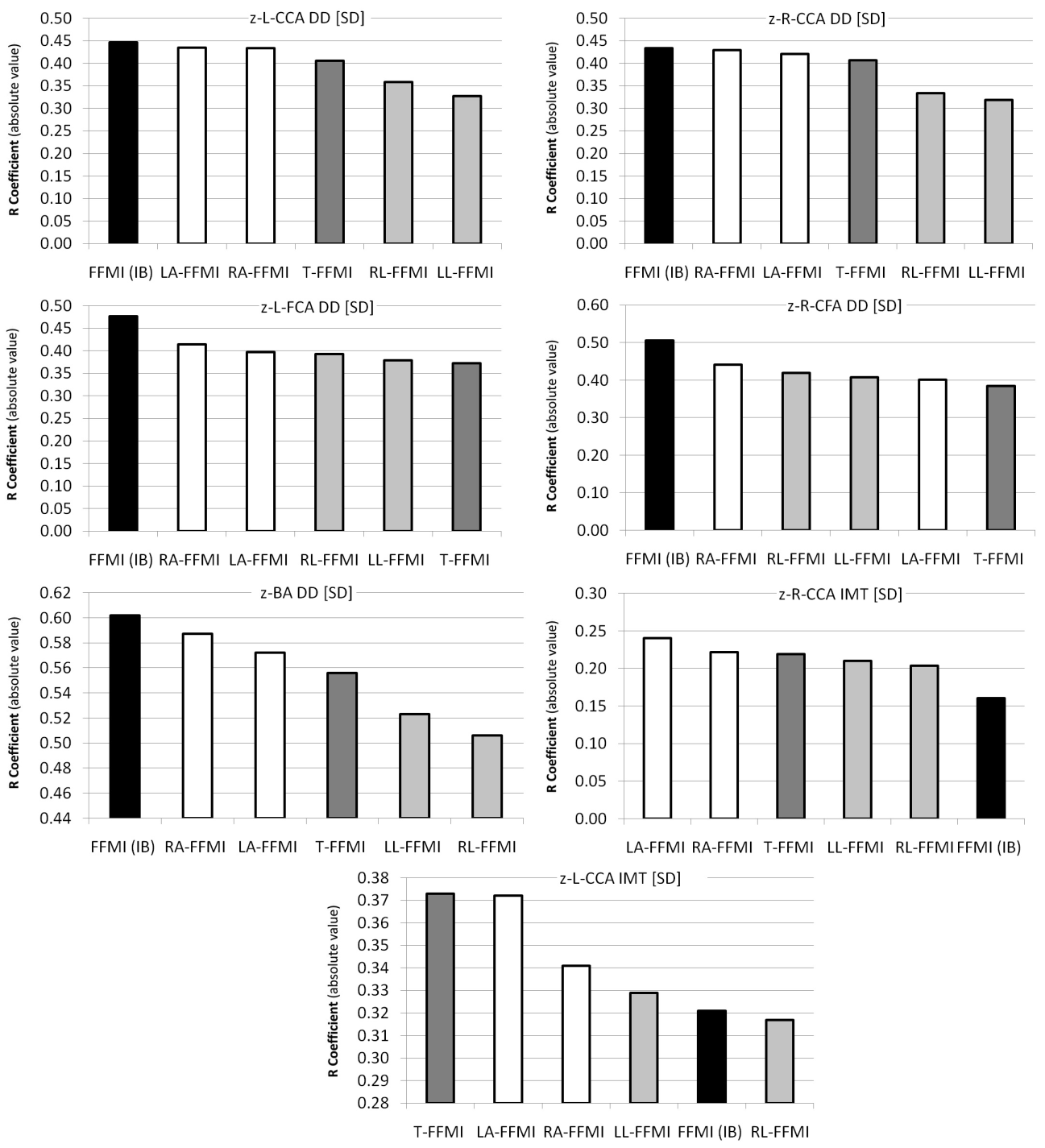


**Figure S6.** Comparison of the levels of association (´r´, absolute value ranked from highest to lowest) between structural z-scores (diameters and IMT) and FFMI levels obtained for: (i) whole body (FFMI[IB]), (ii) trunk (T-FFMI), (iii) right and left upper limbs (RA-FFMI, LA-FFMI), and (iv) right and left lower limbs (RL-FFMI, LL-FFMI). Abbreviations as in text.


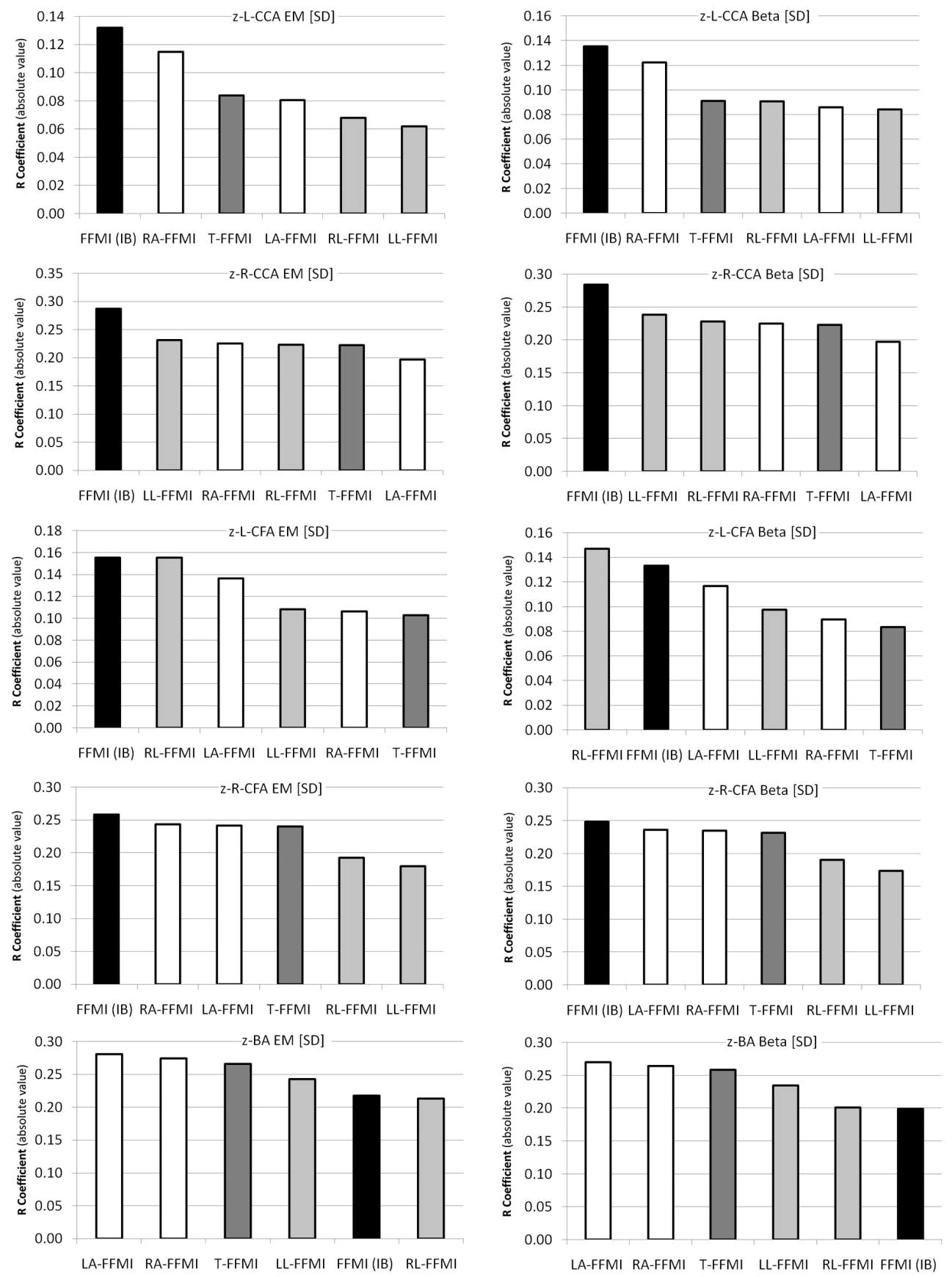


**Figure S7.** Comparison of the levels of association (´r´, absolute value ranked from highest to lowest) between local stiffness z-scores (EM and β) and FFMI levels obtained for: (i) whole body (FFMI[IB]), (ii) trunk (T-FFMI), (iii) right and left upper limbs (RA-FFMI, LA-FFMI), and (iv) right and left lower limbs (RL-FFMI, LL-FFMI). Abbreviations as in text.


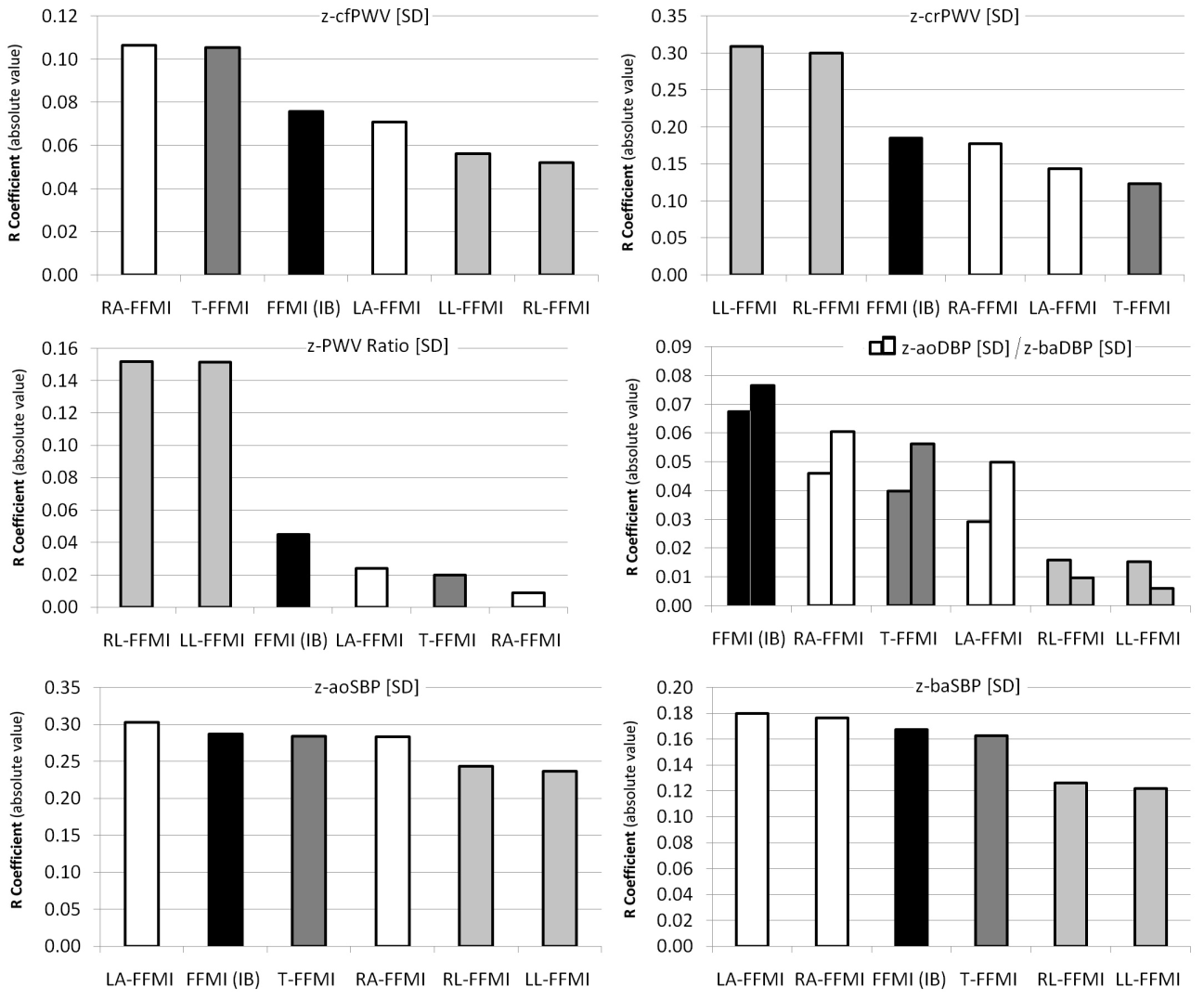


**Figure S8**. Comparison of levels of association (´r´, absolute value ranked from highest to lowest) between regional stiffness z-scores, BP z-scores and FFMI levels obtained for: (i) whole body (FFMI[IB]), (ii) trunk (T-FFMI), (iii) right and left upper limbs (RA-FFMI, LA-FFMI), and (iv) right and left lower limbs (RL-FFMI, LL-FFMI). Abbreviations as in text.
